# The preferential transport of NO_3_^−^ by full-length *Guillardia theta* anion channelrhodopsin 1 is enhanced by its extended cytoplasmic domain

**DOI:** 10.1016/j.jbc.2023.105305

**Published:** 2023-09-29

**Authors:** Yuya Ohki, Tsukasa Shinone, Sayo Inoko, Miu Sudo, Makoto Demura, Takashi Kikukawa, Takashi Tsukamoto

**Affiliations:** 1 Division of Soft Matter, Graduate School of Life Science, Hokkaido University, Sapporo, Japan; 2 Division of Macromolecular Functions, Department of Biological Science, School of Science, Hokkaido University, Sapporo, Japan; 3 Faculty of Advanced Life Science, Hokkaido University, Sapporo, Japan

**Keywords:** anion transport, intrinsically disordered protein, ion channel, kinetics, membrane protein, photobiology, photoreceptor, protein expression, rhodopsin, transmembrane domain

## Abstract

Previous research of anion channelrhodopsins (ACRs) has been performed using cytoplasmic domain (CPD)-deleted constructs and therefore have overlooked the native functions of full-length ACRs and the potential functional role(s) of the CPD. In this study, we used the recombinant expression of full-length *Guillardia theta* ACR1 (*Gt*ACR1_full) for pH measurements in *Pichia pastoris* cell suspensions as an indirect method to assess its anion transport activity and for absorption spectroscopy and flash photolysis characterization of the purified protein. The results show that the CPD, which was predicted to be intrinsically disordered and possibly phosphorylated, enhanced NO_3_^−^ transport compared to Cl^−^ transport, which resulted in the preferential transport of NO_3_^−^. This correlated with the extended lifetime and large accumulation of the photocycle intermediate that is involved in the gate-open state. Considering that the depletion of a nitrogen source enhances the expression of *Gt*ACR1 in native algal cells, we suggest that NO_3_^−^ transport could be the natural function of *Gt*ACR1_full in algal cells.

Transmembrane α-helical proteins play vital roles in fundamental biological processes in living organisms. They are involved in the transportation of ions and small molecules, in cellular signal transduction, in enzymatic reactions, and so on. Microbial rhodopsins are a family of such proteins and they function in response to light. In the last 2 decades, a significant number of microbial rhodopsins have been discovered and, at the same time, the diversity of their light-dependent molecular functions has been clarified, such as ion pumps, ion channels, light sensors, enzymes, and so on (1).

Microbial rhodopsins are commonly composed of seven transmembrane α-helices and a chromophore all-*trans*-retinal which covalently binds to a conserved Lys residue in the seventh transmembrane helix (1). This has come to be called the “rhodopsin domain” (Fig. 1*A*), which has a length of approximately 240 to 300 amino acid residues and thus a molecular mass of 26 to 33 kDa. Most microbial rhodopsins, such as ion pump and phototaxis sensor-type rhodopsins, have only the rhodopsin domain (Fig. 1*A*, left). However, enzyme- and ion channel-type rhodopsins are different. The enzyme rhodopsins are composed of the rhodopsin domain, an additional transmembrane α-helix attached to the N terminus of the rhodopsin domain, and the enzyme domain attached to the C terminus of the rhodopsin domain, which is located inside the cytoplasmic side of the cell membrane (Fig. 1*A*, center) (2). Ion channel rhodopsins, called channelrhodopsins (ChRs), have the rhodopsin domain and an extended cytoplasmic domain (CPD) attached to the C terminus of the rhodopsin domain, which protrudes into the cytoplasm (Fig. 1*A*, right) (3). Unlike the enzyme domain of enzyme rhodopsins, little is known about the functional role(s) of the CPD in ChRs since the discoveries of ChRs for cations (cation channelrhodopsins, CCRs) (4, 5, 6) and for anions (anion channelrhodopsins, ACRs) (6, 7). The size of the CPD is different for each protein and in general, the CPD in CCRs (around 200–400 amino acids long) is larger than in ACRs (around 100–200 amino acids long) (Fig. 1*B*).

**Figure 1:**
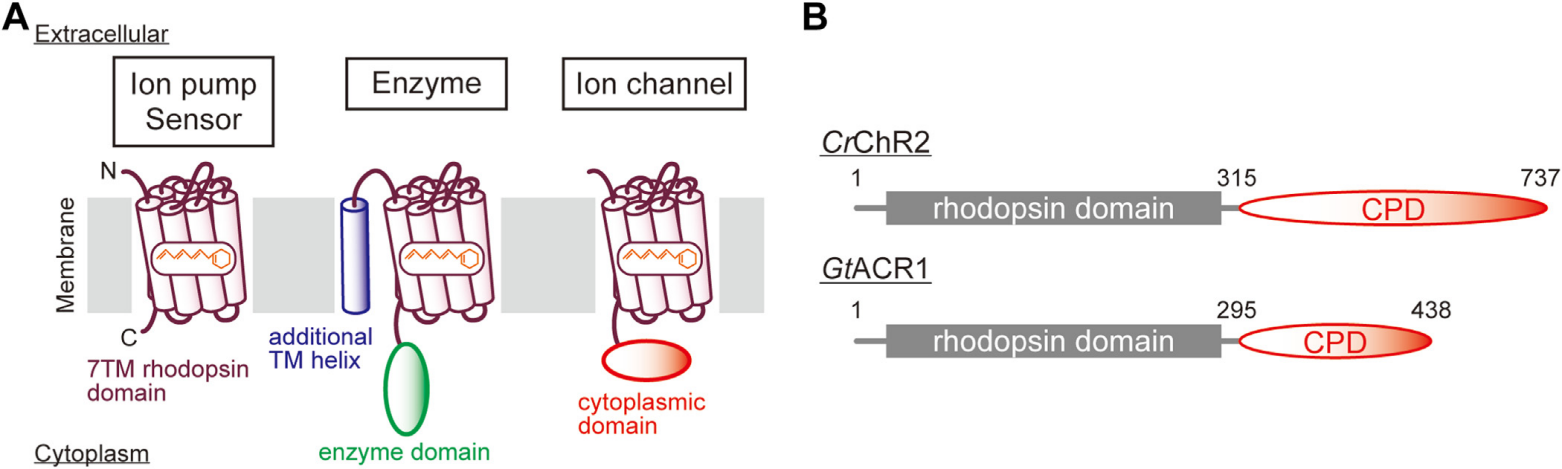
**Illustrations for the structures of microbial rhodopsins.***A*, structural illustrations of microbial ion pump and light sensor (*left*), enzyme (*center*), and ion channel (*right*) rhodopsins. In addition to the 7-transmembrane (TM) rhodopsin domain for each protein, the additional TM helix (*blue*), the cytoplasmic enzyme domain (*green*), and the extended cytoplasmic domain (CPD, *red*) are shown. *B*, comparison of the full-length primary sequences of *Chlamydomonas reinhardtii* cation channelrhodopsin 2 (*Cr*ChR2) and *Guillardia theta* anion channelrhodopsin 1 (*Gt*ACR1), which are representative CCRs and ACRs, respectively. ACR, anion channelrhodopsin; CCR, cation channelrhodopsin.

Up until now, many research groups have investigated CPD-deleted ChRs because deletion of the CPD results in increased protein expression levels, and CCRs and ACRs exhibit ion channel activities even in the absence of the CPD. Therefore, the original molecular functions and biological roles of full-length ChRs, as well as the potential functional role(s) of the CPD, have been overlooked. Nevertheless, for CCRs that originate from the green alga *Chlamydomonas reinhardtii*, it was shown that the CPD did not contribute to the cation transport (8) but was involved in membrane localization and protein–protein interactions in native algal cells (9). Moreover, a recent study by Tashiro *et al.* (10) demonstrated that the CPD of KnChR, a CCR discovered in the green alga *Klebsormidium nitens*, contributes to the cation transport function possibly through an interaction with the rhodopsin domain. On the other hand, the functional role of the CPD in ACRs remains unknown.

What is the original function of full-length ACRs in nature? How is that achieved at the molecular level? What is the functional role of the CPD in ACRs? To answer those questions, in this study, we prepared full-length *Gt*ACR1 (*Gt*ACR1_full, Fig. 1*B*), a well-studied ACR from a cryptophyte alga *Guillardia theta*, using a recombinant expression system. Then, we characterized the anion transport function of *Gt*ACR1_full to identify its original function. We successfully constructed a recombinant expression system for *Gt*ACR1_full using the yeast *Pichia pastoris*. Using that expression system and the pH electrode method that we reported previously (11, 12), we found that the anion transport activity of *Gt*ACR1_full was generally weaker than *Gt*ACR1 without the CPD (*Gt*ACR1_ΔCPD). However, *Gt*ACR1_full showed a significantly enhanced transport preference for NO_3_^−^. This result indicated that the CPD has an inhibitory effect on the intensity of anion transport activity but contributes to the development of anion preference by some mechanism. To reveal the mechanism involved at the molecular level, we analyzed the photoreaction cycle, which is called the photocycle and is directly connected to the anion transport function. As a result, the preferential transport of NO_3_^−^ by *Gt*ACR1_full was considered to result from the extended lifetime, the large accumulation of the photocycle intermediate involved in the gate-open state and the increase in specific efficiency for NO_3_^−^ against Cl^−^, which were provided by the CPD. Based on these results, we considered the biological role of *Gt*ACR1_full in native *G. theta* in terms of the preferential transport of NO_3_^−^. Furthermore, we hypothesized that the CPD contributes to the preferential transport of NO_3_^−^ possibly through an interaction not only with NO_3_^−^ but also with the rhodopsin domain. Indeed, it may be easier to conduct experiments if the CPD is deleted but this study has finally begun to show what could only be seen with full-length ACRs in which the CPD has not been deleted.

## Results

### The CPD is intrinsically disordered and is possibly phosphorylated

As shown in Figure 1, CCRs and ACRs have extended CPDs composed of approximately 200 to 400 amino acid residues in CCRs and approximately 100 to 200 amino acid residues in ACRs. Fig. S1 shows the amino acid sequence alignment of the 7-transmembrane rhodopsin domains of several ACRs as reported previously (7, 13, 14). The residue numbers listed correspond to the *Gt*ACR1 sequence (7). Functionally important amino acid residues are well conserved, such as Cys21 and Cys219, which form an intramolecular disulfide bridge (15, 16), Lys238 where the chromophore all-*trans*-retinal binds *via* a protonated Schiff base linkage, Glu68 and Asp234, which are located near the protonated retinal Schiff base, and Asn239. On the other hand, there is no overall conservation of amino acid sequences of CPDs in ACRs, as shown in Fig. S2. In addition, no known functional domains, such as a peptidoglycan-binding domain FimV in KnChR (10), are found in most of the sequences.

A previous study on CCRs derived from *Volvox carteri*, named VChR1 (837 residues in total) and VChR2 (747 residues in total), reported that in their long CPDs (450–540 residues), there were three highly conserved regions, named con1, con2, and con3, respectively (17). However, the sequences of the CPDs in *Gt*ACR1 and *Gt*ACR2 are far shorter (140–150 residues) and share overall high sequential homology with each other (identity 36%; similarity 76%) (Fig. S3). Therefore, there is no con region in the CPDs of *Gt*ACRs as same as that in VChRs. Rather, the entire CPDs is the con region in the case of *Gt*ACRs. In addition, the con1, con2, and con3 sequences in VChRs are not conserved in *Gt*ACRs. The same previous study reported that there were significantly more Met-Gly and Asn-Gly repeat sequences in the CPDs of VChRs (80 and 15 repeats in total for VChR1 and VChR2, respectively) (17). However, in the CPDs of *Gt*ACRs, there is no such kind of noticeably more repetitive sequences. At most, there observed five repeats of Asp-Ser, Ser-Asp for *Gt*ACR1, and five repeats of Ser-Glu for *Gt*ACR2, respectively. The CPDs of both *Gt*ACRs contain high percentages of Lys/Arg (27.3% for *Gt*ACR1 and 27.1% for *Gt*ACR2) and Asp/Glu (20.3% for *Gt*ACR1 and 20.7% for *Gt*ACR2) and therefore have positive charge at a neutral pH. In addition, Ser is the third most contained residue (11.2% for *Gt*ACR1 and 11.6% for *Gt*ACR2), which may relate to potential phosphorylation of the CPDs as described below.

**Figure 2:**
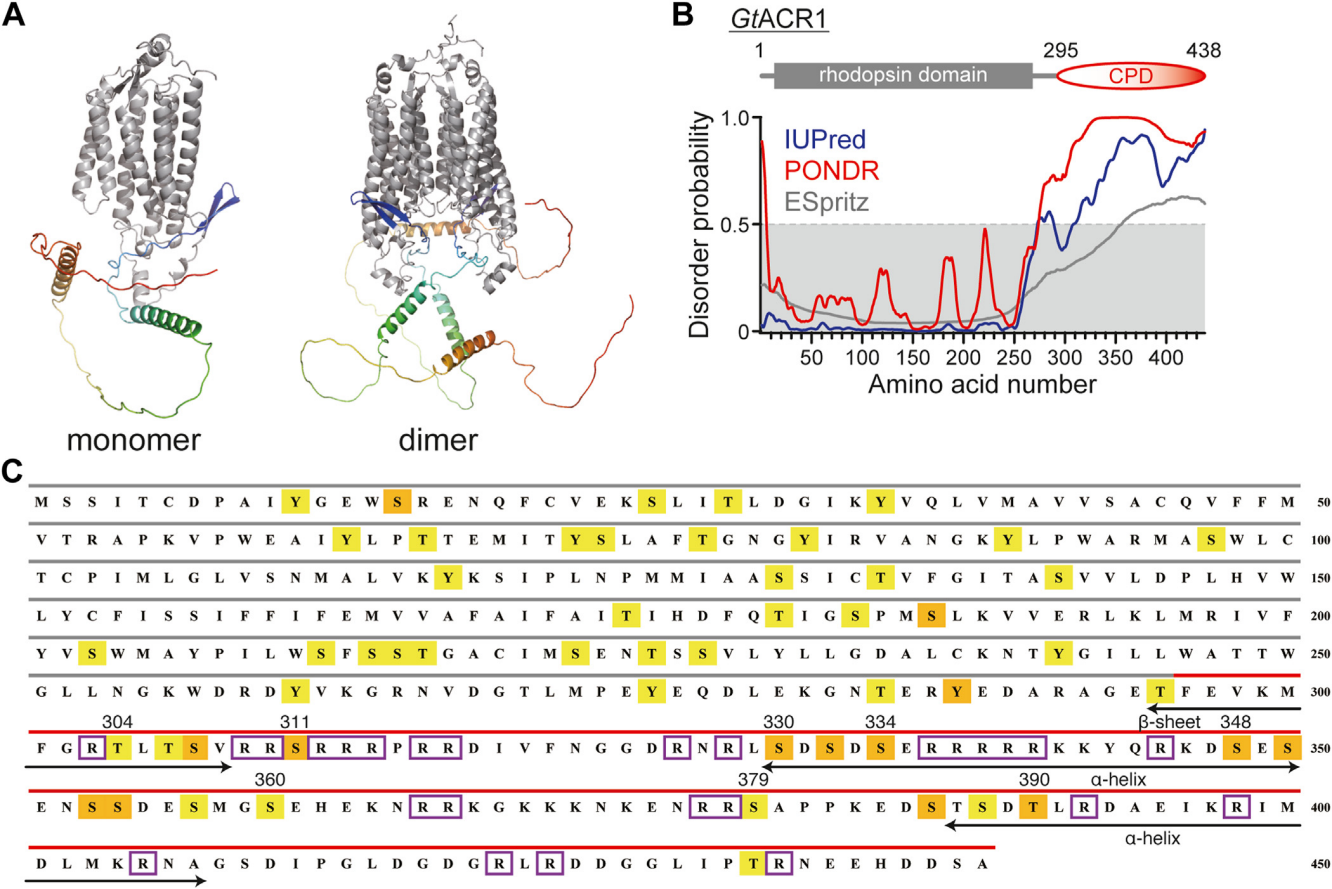
**Structural predictions of full-length *Gt*ACR1.***A*, three-dimension structural models for monomeric (*left*) and dimeric (*right*) *Gt*ACR1_full predicted by AlphaFold2. *B*, predictions of structural disorderness for *Gt*ACR1_full according to IUPred (*blue*), PONDR (*red*), and ESpritz (*gray*) programs. *C*, predictions of phosphorylation sites (Ser, Thr, and Tyr residues) for *Gt*ACR1_full performed by the NetPhos 3.1 program. *Orange* and *yellow rectangles* indicate possible phosphorylation sites more than 95% and 50%, respectively. *Gray* and *red* bars above the sequence indicate the rhodopsin domain and the CPD, respectively. Arginine residues in the CPD are indicated by *purple* boxes. Secondary structures α-helix and β-sheet, which are predicted by AlphaFold2 (*panel A*), are indicated by *black arrows*. ACR, anion channelrhodopsin; CPD, cytoplasmic domain.

We constructed model structures of *Gt*ACR1_full monomers and dimers using AlphaFold2 (Fig. 2*A*) (18) by referring to X-ray crystallographic structures of *Gt*ACR1_ΔCPD solved as dimers (15, 16, 19) as in *Cr*ChR2 (20, 21). As a result, we obtained disordered structures of the CPD independent from monomer or dimer. Some secondary structures were partially predicted as shown in Figure 2, *A* and *C*. We then applied the amino acid sequence of *Gt*ACR1_full to prediction programs named IUPred (22), PONDR (23), and ESpritz (24) to analyze the disorderness of the CPD (Fig. 2*B*). As a result, the CPD was predicted to be intrinsically disordered (the disorder probability was more than 0.5 (50%)).

In general, intrinsically disordered proteins/regions are common in eukaryotes, including alga, and share significantly poor amino acid conservation (25, 26). This was the case for the CPDs of ACRs as shown in Fig. S2. In addition, the intrinsically disordered proteins/regions are capable of binding or interacting with a variety of substrates, which are coupled with protein folding and have the characteristic of flexibly changing their folding structure to match the substrate (27). In the case of membrane proteins, there tends to be more intrinsically disordered regions on the cytoplasmic side (28).

*C. reinhardtii* ChR1 (*Cr*ChR1) has multiple phosphorylation sites in its sequence both in the rhodopsin domain and in the CPD (29). Phosphorylation has been shown to regulate the phototactic behavior of *C. reinhardtii* in different light conditions, which allows us to expect that the cation transport function of *Cr*ChR1 is modulated by phosphorylation. We used the NetPhos 3.1 program (30) to identify possible phosphorylation sites in *Gt*ACR1_full and found multiple predicted phosphorylation sites, Ser, Thr, and Tyr, throughout the sequence (Fig. 2*C*). Especially in the CPD (the region indicated by the red bar), more highly scored (more than 95%) candidates were obtained than in the rhodopsin domain (highlighted in orange). Previous study on *Cr*ChR1 identified 10 phosphorylation sites in its CPD using mass spectrometry (29). There were three phosphorylation sites near the rhodopsin domain and the rest of seven sites was clustered at the C-terminal region in the CPD. On the other hand, 18 potential phosphorylation sites were predicted in the CPD of *Gt*ACR1 (Fig. 2*C*). They are roughly divided into 4 clusters (Thr304 – Ser311, Ser330 – Ser334, Ser348 – Ser360, and Ser379 – Thr390) and many of the candidates were predicted to exist in the regions that form secondary structures. Unfortunately, the commonality and difference in the phosphorylation between *Cr*ChR1 and *Gt*ACR1 are unclear at present.

In this study, we focused on *Gt*ACR1 (7), which has been the most studied ACR, for recombinant preparation and various analyses to clarify the molecular function of *Gt*ACR1_full compared with *Gt*ACR1_ΔCPD. Then, we discuss the biological role of *Gt*ACR1_full and the functional role of the CPD.

### Recombinant preparation of the full-length *Gt*ACR1

**Figure 3:**
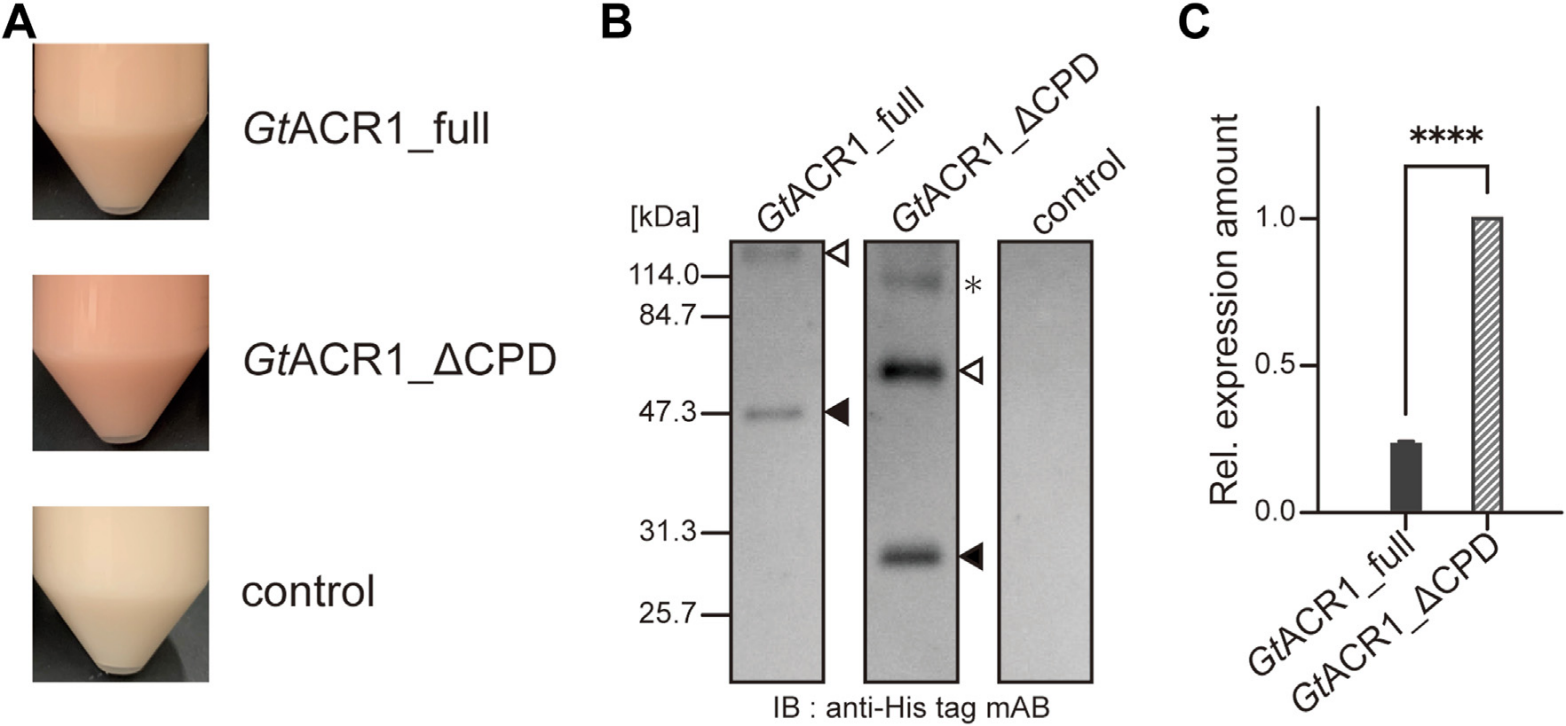
**Recombinant expression of full-length *Gt*ACR1.***A*, image of *Pichia pastoris* cells expressing *Gt*ACR1_full (*top*) and *Gt*ACR1_ΔCPD (*middle*); cells without the transforming *Gt*ACR1 gene are shown on the *bottom* as a negative control. *B*, images of SDS-PAGE and Western blotting of *Gt*ACR1_full (*left*), *Gt*ACR1_ΔCPD (*center*), and the negative control (*right*). Immunoblotting was done using an anti-His tag mono-antibody. *White* and *black triangles* indicate dimer and monomer bands, respectively; the *asterisk* indicates aggregates. *C*, statistical comparison of the expression level of *Gt*ACR1_full (*left*, *solid black bar*) and *Gt*ACR1_ΔCPD (*right*, *striped bar*) estimated in *panel B* using ImageJ software (64). Data are reported as means and S.D. (*n* = 3). For *Gt*ACR1_full, the mean ± S.D. is 2.42 × 10^−1^ ± 6.39 × 10^−3^. An unpaired *t* test was performed (*p*-value; ∗∗∗∗ < 0.0001). ACR, anion channelrhodopsin; CPD, cytoplasmic domain.

As described in the Experimental procedures section, we selected the yeast *P. pastoris* recombinant expression system and incorporated the gene for *Gt*ACR1_full into the pPICZ B vector after codon optimization. The *P. pastoris* system has often been used for recombinant expression not only for ACRs but also for CCRs. With the expectation of increased and visible expression of *Gt*ACR1_full, we tried to obtain multi-copy recombinants of transformed *P. pastoris* by seeding on YPDS (yeast extract, peptone, dextrose, and sorbitol medium) agar plates including 100 to 2000 μg antibiotics Zeocin. As a result, by comparing to the negative control without incorporation of the ACR gene, red-colored *P. pastoris* cells were obtained, indicating that the functional expression of *Gt*ACR1_full had succeeded (Fig. 3*A*, top). However, as expected, the red color of cells expressing *Gt*ACR1_full was weaker than cells expressing *Gt*ACR1_ΔCPD (Fig. 3*A*, middle), indicating that the increased expression of *Gt*ACR1_ΔCPD is due to deletion of the CPD.

To confirm the protein expression level and estimate that quantitatively, SDS-PAGE and Western blotting were performed (Fig. 3*B*). As a result, *Gt*ACR1_full was detected as two bands near the 47.3 kDa and 114.0 kDa molecular markers compared to the negative control. Because the calculated molecular mass of *Gt*ACR1_full is 51.0 kDa, the smaller and the larger bands corresponded to the monomer (indicated by the black triangle) and the dimer (white triangle), respectively. On the other hand, in the case of *Gt*ACR1_ΔCPD, which has a calculated molecular mass of 34.4 kDa, three bands were detected, which were assigned as the monomer (black triangle), the dimer (white triangle), and aggregates (asterisk). From the total band intensity, the relative protein expression levels (Fig. 3*C*) were estimated and the expression level of *Gt*ACR1_full was about 24% of *Gt*ACR1_ΔCPD.

### *Gt*ACR1_full preferentially transports NO_3_^−^

Previously, we measured anion transport activity using a pH electrode method (12). For that measurement, the pH electrode was placed into a suspension of *Gt*ACR1-expressing *P. pastoris* cells. Light activates *Gt*ACR1, which results in the influx of anions through the protein because the anion concentration was adjusted to be higher outside of the cells (300 mM) than inside the cells. This anion influx induces the penetration of H^+^ from outside to inside the cells to compensate for the transiently increased negative membrane potential. Therefore, the pH electrode method can indirectly detect the anion transport activity of ACRs. Here, we measured the transport activities of *Gt*ACR1_full for various anions, including F^−^, Cl^−^, Br^−^, I^−^, NO_3_^−^, SO_4_^2−^, and aspartate (Asp^-^), using the pH electrode method. We also measured the transport activities of *Gt*ACR1_ΔCPD of those anions for comparison.

**Figure 4:**
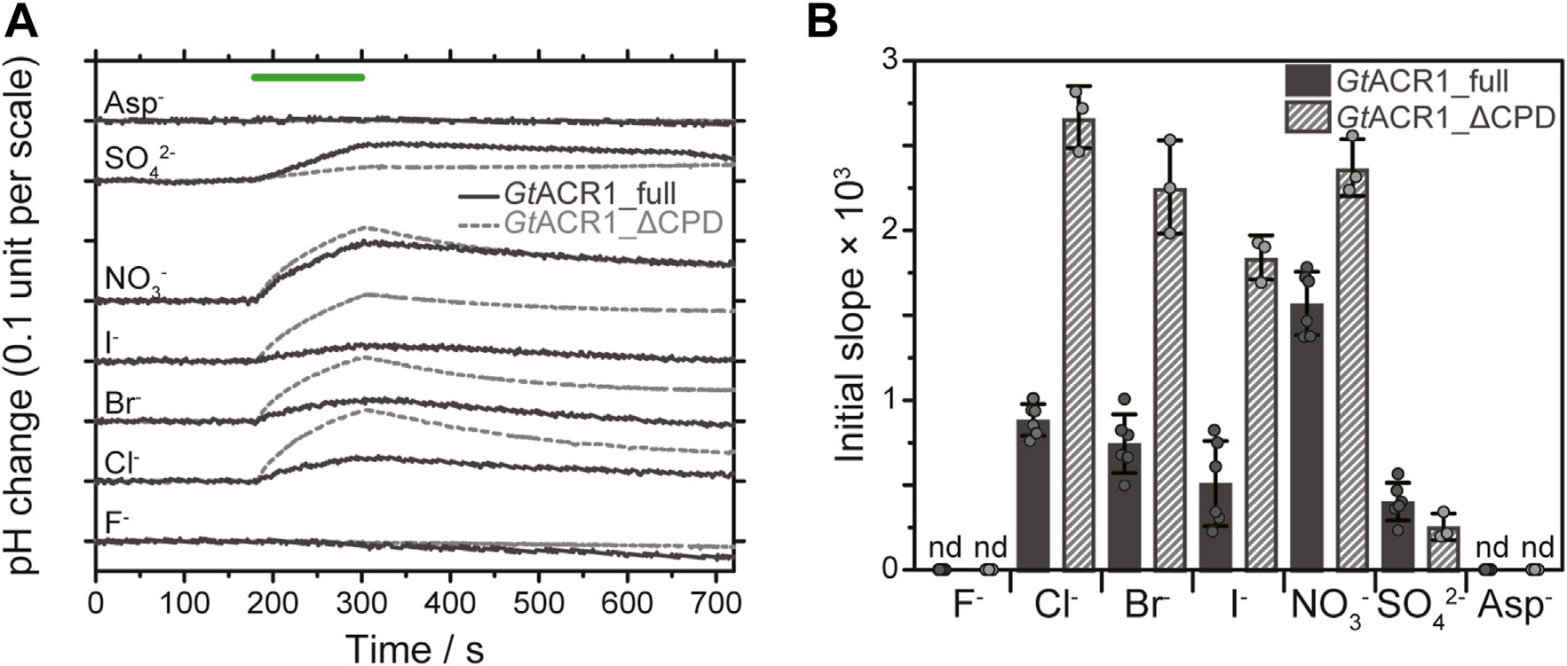
**Anion transport activity measurement using the pH electrode method.** The data for *Gt*ACR1_full were corrected by protein expression levels estimated from Western blotting (Fig. 3*C*). *A*, time-dependent pH changes of *Pichia pastoris* suspensions expressing *Gt*ACR1_full (*black solid lines*) and *Gt*ACR1_ΔCPD (*gray dotted lines*). Each sodium salt was dissolved at a concentration of 300 mM. *Green* LED light (530 nm) was illuminated for 2 min as indicated by the *green* bar. *B*, comparisons of anion transport activities of *Gt*ACR1_full (*solid black bars*; *n* = 6) and *Gt*ACR1_ΔCPD (*striped bars*; *n* = 3). The anion transport activities were calculated by the initial slope of the first 10 s after LED light illumination for time-dependent pH changes (*panel A*). Data are reported as means ± S.D. “nd” means “not detected”. For *Gt*ACR1_full, the mean ± S.D. values are 8.76 × 10^−4^ ± 9.35 × 10^−5^ for Cl^−^, 7.38 × 10^−4^ ± 1.71 × 10^−4^ for Br^−^, 5.05 × 10^−4^ ± 2.49 × 10^−4^ for I^−^, 1.56 × 10^−3^ ± 1.85 × 10^−4^ for NO_3_^−^, and 3.98 × 10^−4^ ± 1.10 × 10^−4^ for SO_4_^2−^, respectively. For *Gt*ACR1_ΔCPD, the mean ± S.D. values are 2.64 × 10^−3^ ± 1.81 × 10^−4^ for Cl^−^, 2.24 × 10^−3^ ± 2.72 × 10^−4^ for Br^−^, 1.83 × 10^−3^ ± 1.28 × 10^−4^ for I^−^, 2.35 × 10^−3^ ± 1.66 × 10^−4^ for NO_3_^−^, and 2.50 × 10^−4^ ± 7.73 × 10^−5^ for SO_4_^2−^, respectively. No statistical analysis was performed. ACR, anion channelrhodopsin; CPD, cytoplasmic domain.

Figure 4*A* shows the time-dependent pH changes originating from the transport activities of *Gt*ACR1_full (black solid lines) and *Gt*ACR1_ΔCPD (gray dotted lines) in the presence of various anions. The data for *Gt*ACR1_full were corrected by the protein expression level estimated from Western blotting (Fig. 3*C*). Figure 4*B* summarizes the initial slope amplitudes calculated from the data shown in Figure 4*A*. These results clearly show that the anion transport activities of *Gt*ACR1_full were smaller in general than those of *Gt*ACR1_ΔCPD, except for SO_4_^2−^. The initial slope amplitudes of *Gt*ACR1_full for Cl^−^, Br^−^, and I^−^ were decreased to nearly one-third compared to *Gt*ACR1_ΔCPD. However, the initial slope amplitude of *Gt*ACR1_full in the presence of NO_3_^−^ was about two-thirds compared to *Gt*ACR1_ΔCPD and therefore was more than about 2-times larger than that of *Gt*ACR1_full in the presence of Cl^−^, Br^−^, and I^−^. A previous patch clamp analysis of *Gt*ACR1_ΔCPD expressed in mammalian cells showed that its relative permeability for NO_3_^−^ was higher than that for Cl^−^, Br^−^, and I^−^ (7), and a similar result was obtained using our pH electrode method (12). The results of this study revealed that *Gt*ACR1_full also significantly and preferentially transports NO_3_^−^ compared to the other anions. Such an anion transport preference has been reported for MerMAIDs (31) but not for Prasinophyte and viral ACRs (32). As the pH-electrode method is less quantitative than the patch clamp method, future work will be required for further detailed quantitative analysis of the anion transport activity for *Gt*ACR1_full.

### Basic photochemical properties of purified *Gt*ACR1_full

To investigate the functional characteristics of *Gt*ACR1_full at the molecular level, we purified *Gt*ACR1_full in the presence of a detergent, *n*-dodecyl-β-D-maltopyranoside (DDM). Here, we set two goals, which were to reveal: (1) what the basic photochemical properties of *Gt*ACR1_full are compared with *Gt*ACR1_ΔCPD and (2) why *Gt*ACR1_full preferentially transports NO_3_^−^.

**Figure 5:**
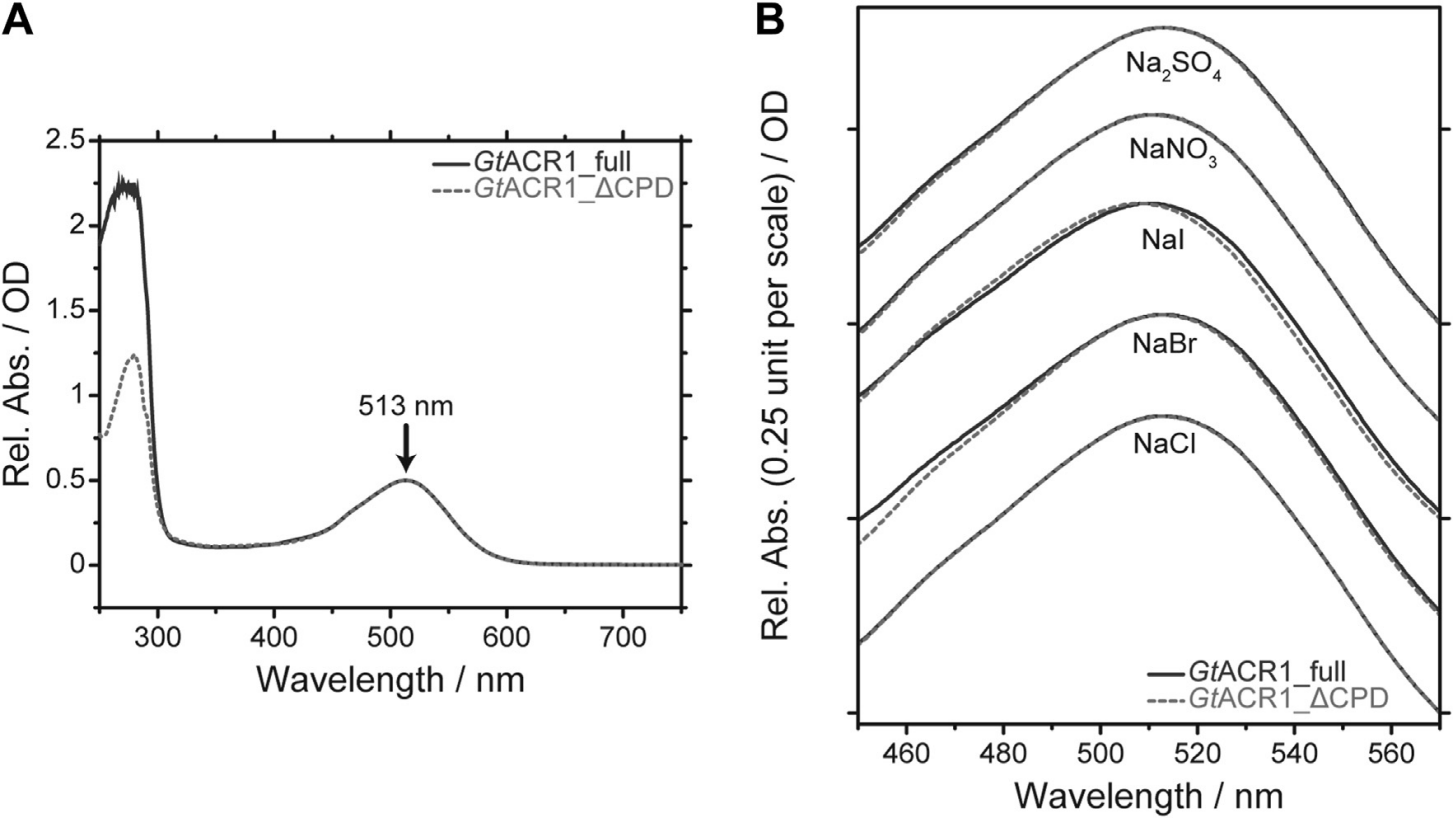
**Initial state spectroscopic properties of purified *Gt*ACR1_full (*black solid line*) and *Gt*ACR1_ΔCPD (*gray dotted line*).***A*, UV-visible absorption spectra in the presence of 1 M NaCl. The λ_max_ was 513 nm indicated by an arrow. Absorbance is shown as a relative value. *B*, comparisons of visible absorption spectra in the presence of various salts at concentrations of 1 M. ACR, anion channelrhodopsin; CPD, cytoplasmic domain.

For the first goal, we measured UV-visible absorption spectra (Fig. 5) to investigate the initial state property. In the presence of Cl^−^, the spectra of *Gt*ACR1_full (black solid line) and *Gt*ACR1_ΔCPD (gray dotted line) were identical in the visible region and thus exhibited the same maximum absorption wavelength (λ_max_) at 513 nm (Fig. 5*A*). On the other hand, in the UV region, the absorption of *Gt*ACR1_full was larger than *Gt*ACR1_ΔCPD due to the additional CPD, which contains one Tyr and three Phe residues (Fig. S2) and an unexpected impurity. To determine the anion-dependent spectral changes, the visible absorption spectra of *Gt*ACR1_full and *Gt*ACR1_ΔCPD were measured in the presence of several anions (Fig. 5*B*). As a result, anion-dependent visible spectral changes were hardly observed for either protein. Therefore, these results indicate that the initial state properties of *Gt*ACR1_full and *Gt*ACR1_ΔCPD are identical, meaning that the CPD does not affect the initial state property.

**Figure 6:**
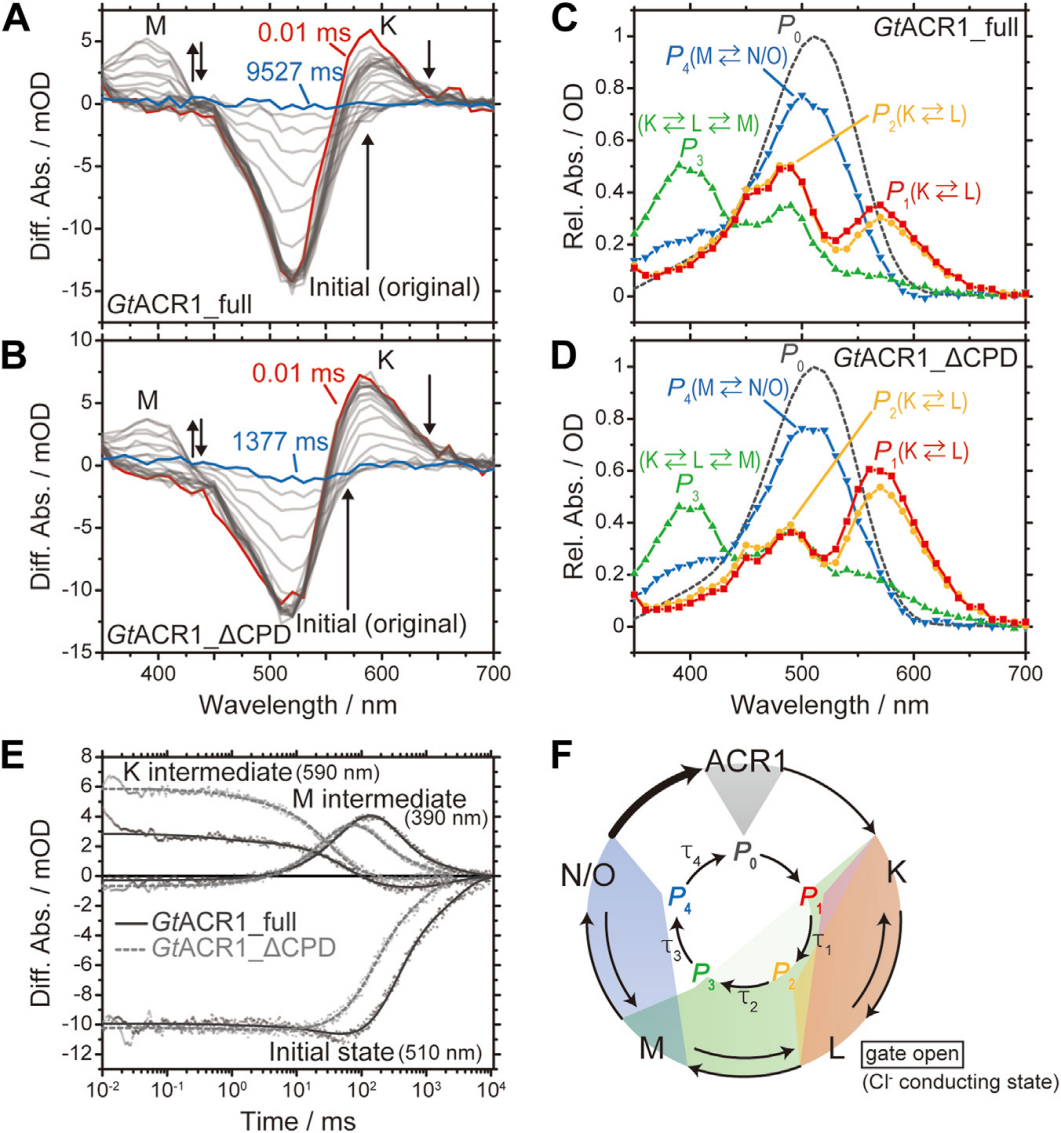
**Photoreaction properties in the presence of 1 M NaCl.***A* and *B*, flash-induced light-minus-dark difference absorption spectra of (*A*) *Gt*ACR1_full and (*B*) *Gt*ACR1_ΔCPD. Absorption changes are indicated by arrows. *C* and *D*, absorption spectra of kinetically distinguished *P*_i_ (*i* = 1–4) states for (*C*) *Gt*ACR1_full and (*D*) *Gt*ACR1_ΔCPD. The spectrum of *P*_0_ (*dotted line*) is that of the initial state. The names of photo-intermediates in each *P*_i_ state are described. Absorbance is shown as a relative value. *E*, transient absorption changes of *Gt*ACR1_full (*black*) and *Gt*ACR1_ΔCPD (*gray*). The raw data are shown as dots and fitting curves are shown as lines (the curve of *Gt*ACR1_ΔCPD is shown as a *dotted line*). The wavelengths corresponding to the initial state (510 nm), K (590 nm), and M (390 nm) are presented. *F*, photocycle scheme of *Gt*ACR1_full and *Gt*ACR1_ΔCPD based on the kinetic analysis. The L-intermediate is the gate-open (Cl^−^-conducting) state (33, 37). The difference of the photocycle between *Gt*ACR1_full and *Gt*ACR1_ΔCPD is highlighted as a *bold black arrow*. ACR, anion channelrhodopsin; CPD, cytoplasmic domain.

We then measured transient absorption changes using the flash photolysis method. That method can analyze the kinetic behavior of photo-intermediates during the photocycle, which is directly connected to the anion transport function. First, we compared the photocycles of *Gt*ACR1_full and *Gt*ACR1_ΔCPD in the presence of Cl^−^ to represent the anions used in this study. Figure 6, *A* and *B* show the flash-induced difference absorption spectra of *Gt*ACR1_full and *Gt*ACR1_ΔCPD, respectively. As previously reported, after excitation by laser flash, *Gt*ACR1_ΔCPD showed absorption changes at 510 nm (negative band, -), 390 nm (positive band, +), and 590 nm (+), which are assigned as the initial state, the M-intermediate, and the K-intermediate, respectively (33). These absorption changes were reproduced, as shown in Figure 6*B*. On the other hand, the same absorption changes at 510 nm (−), 390 nm (+), and 590 nm (+) were observed in the case of *Gt*ACR1_full (Fig. 6*A*), which indicates that *Gt*ACR1_full shares the same photo-intermediates with *Gt*ACR1_ΔCPD during the photocycle. Figure 6, *C* and *D* represent the calculated absorption spectra of the initial state, *P*_0_, and four kinetic states, *P*_1_ – *P*_4_, in *Gt*ACR1_full and *Gt*ACR1_ΔCPD, respectively. As a result of spectral separation (see details in Figs. S4 and S5) and by referring to previous studies, the compositions of the photo-intermediates for *Gt*ACR1_full were the K- and L-intermediates in equilibrium in the *P*_1_ and *P*_2_ states, the K-, L-, and M-intermediates in equilibrium in the *P*_3_ state, and the M- and N/O-intermediates in equilibrium in the *P*_4_ state, respectively (Fig. 6*C*). This is the same as for *Gt*ACR1_ΔCPD (Fig. 6*D*).

However, a difference in the photocycle kinetics was found between *Gt*ACR1_full and *Gt*ACR1_ΔCPD in the presence of Cl^−^ (Fig. 6*E*), showing transient absorption changes at 510 nm (initial state), 390 nm (M-intermediate), and 590 nm (K-intermediate). The time constants analyzed by global fitting are summarized in Table 1. The time constants τ_1_ and τ_2_ were comparable between them. Especially when comparing the time constant τ_4_ for the last fourth transition, which is the process of recovery to the initial state and thus the rate-limiting step, the value for *Gt*ACR1_full (2560 ms) was about three times larger than that for *Gt*ACR1_ΔCPD (878 ms). This result indicates that the photocycle of *Gt*ACR1_full is roughly three times slower than that of *Gt*ACR1_ΔCPD. Therefore, the absorption changes of *Gt*ACR1_full (black lines) and *Gt*ACR1_ΔCPD (gray lines) did not overlap (Fig. 6*E*). Figure 6*F* summarizes the photocycle model of *Gt*ACR1_full and *Gt*ACR1_ΔCPD in the presence of Cl^−^, in which the difference in their photocycle kinetics is highlighted by a bold black arrow.

**Table 1:** Summary of time constants^a^ determined by global fitting analysis

Time constant/ms	*Gt*ACR1_full		*Gt*ACR1_ΔCPD	
	Cl^−^	NO_3_^−^	Cl^−^	NO_3_^−^
τ_1_/ms	0.587 ± 0.0310	19.0 ± 1.65	1.08 ± 0.0440	0.282 ± 0.0243
τ_2_/ms	58.2 ± 0.639	130 ± 6.65	30.4 ± 0.344	54.7 ± 1.26
τ_3_/ms	319 ± 5.36	2570 ± 56.2	136 ± 2.83	1090 ± 31.2
τ_4_/ms	2560 ± 41.8	9750 ± 2030	878 ± 18.4	4850 ± 258
*F*_c_^b^	0.03	0.025	0.03	0.025

a Time constants were shown together with S.D. of the global fitting analysis.

b *F*_c_ is the excitation ratio estimated in the process of flash photolysis analysis (see Experimental procedures in Supporting information).

### Photocycle of *Gt*ACR1_full in the presence of NO_3_^−^

**Figure 7:**
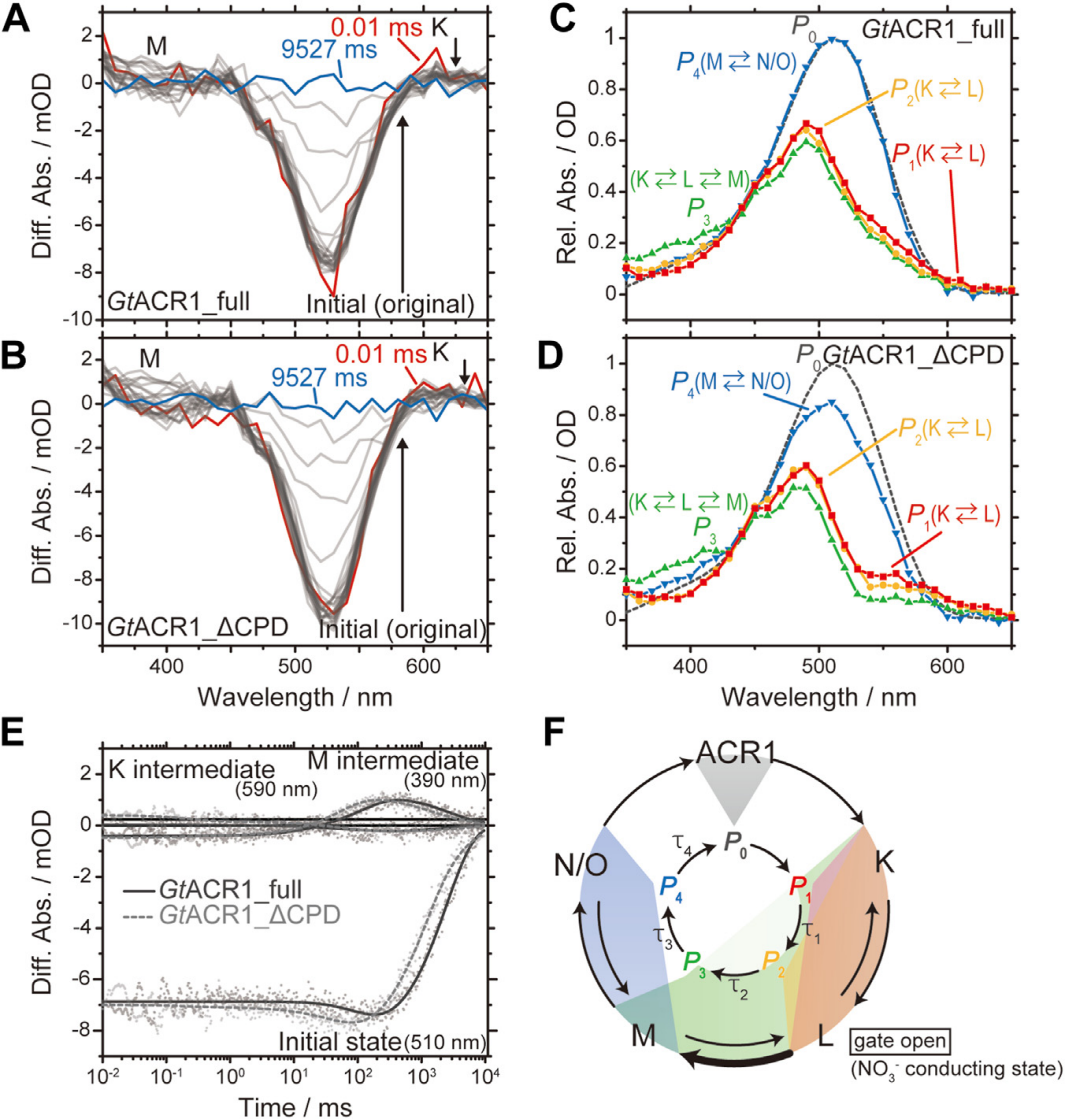
**Photoreaction properties in the presence of 1 M NaNO**_**3**_**.***A* and *B*, flash-induced light-minus-dark difference absorption spectra of (*A*) *Gt*ACR1_full and (*B*) *Gt*ACR1_ΔCPD. Absorption changes are indicated by arrows. *C* and *D*, absorption spectra of kinetically distinguished *P*_i_ (*i* = 1–4) states for (*C*) *Gt*ACR1_full and (*D*) *Gt*ACR1_ΔCPD. The spectrum of *P*_0_ (*dotted line*) is that of the initial state. The names of photo-intermediates in each *P*_i_ state are described. Absorbance is shown as a relative value. *E*, transient absorption changes of *Gt*ACR1_full (*black*) and *Gt*ACR1_ΔCPD (*gray*). The raw data are shown as dots and fitting curves are shown as lines (the curve of *Gt*ACR1_ΔCPD is shown as a *dotted line*). The wavelengths corresponding to the initial state (510 nm), K (590 nm), and M (390 nm) are presented. *F*, photocycle scheme of *Gt*ACR1_full and *Gt*ACR1_ΔCPD based on the kinetic analysis. The gate-open (NO_3_^−^-conducting) state has not yet been determined experimentally. However, in analogy with the case of Cl^−^ (Fig. 6*F*) (33, 37), we estimate that the L-intermediate is also responsible for the gate-open state in the presence of NO_3_^−^. The difference of the photocycle between *Gt*ACR1_full and *Gt*ACR1_ΔCPD is highlighted as a *bold black arrow*. ACR, anion channelrhodopsin; CPD, cytoplasmic domain.

As shown in Figure 4, a significant transport preference for NO_3_^−^ was revealed for *Gt*ACR1_full. For the second goal, we investigated the NO_3_^−^ transport mechanism of *Gt*ACR1_full. Figure 7, *A* and *B* show the flash-induced difference absorption spectra of *Gt*ACR1_full and *Gt*ACR1_ΔCPD, respectively, in the presence of NO_3_^−^. A comparison of the difference absorption spectra in the presence of Cl^−^ (Fig. 6, *A* and *B*) revealed that there was less accumulation of the M- (390 nm) and K- (590 nm) intermediates, which was also supported by the calculated absorption spectra of *P*_0_ – *P*_4_ (Figs. 7, *C* and *D*, S6 and S7). However, our global fitting analysis revealed that both *Gt*ACR1_full and *Gt*ACR1_ΔCPD share basically the same photo-intermediates in the presence of NO_3_^−^. Figure 7*E* shows the transient absorption changes at 510 nm (initial state), 390 nm (M-intermediate), and 590 nm (K-intermediate). We noticed that the photocycle duration in the presence of NO_3_^−^ was extended in both *Gt*ACR1_full and *Gt*ACR1_ΔCPD compared with the presence of Cl^−^ (Fig. 6*E*). Such an anion-dependent delay of the photocycle has been commonly observed in light-driven anion pump halorhodopsins (HRs) (34, 35, 36). Kinetic analysis revealed that each time constant for *Gt*ACR1_full in the presence of NO_3_^−^ was larger than that for *Gt*ACR1_ΔCPD (Table 1). The photocycle duration of *Gt*ACR1_full (9750 ms) was about twice as long as that of *Gt*ACR1_ΔCPD (4850 ms) by comparison to the time constant τ_4_. The photocycle model in the presence of NO_3_^−^ is summarized in Figure 7*F*.

A closer look at the photocycle kinetics data in the presence of Cl^−^ and NO_3_^−^ reveals interesting differences. Here we focused on the time constant for the second transition, τ_2_, which corresponds to generation of the M-intermediate that temporally correlates with the gate-closing process (33, 37) (Figs. 6*F* and 7*F*, and Table 1). In the presence of Cl^−^, the τ_2_ values for *Gt*ACR1_full (58.2 ms) and *Gt*ACR1_ΔCPD (30.4 ms) were similar and thus the rise of the M-intermediate at 390 nm were almost overlapped (Fig. 6*E*). On the other hand, in the presence of NO_3_^−^, the τ_2_ for *Gt*ACR1_full (130 ms) was more than two times larger than that for *Gt*ACR1_ΔCPD (54.7 ms) and thus we observed a delayed generation of the M-intermediate for *Gt*ACR1_full (Fig. 7*E*). This result indicates that the lifetime of the gate-open state, the L-intermediate (33, 37), becomes longer when transporting NO_3_^−^, which is highlighted as a bold black arrow in Figure 7*F*.

## Discussion

All previous research on ACRs have been conducted using CPD-deleted constructs, which still possess anion transport activities upon light illumination. However, the native functions of full-length ACRs and the role(s) of the CPD remained unknown. To resolve those issues, we used a recombinant expression system to express and purify the full-length ACR, *Gt*ACR1_full, and then performed anion transport measurements using the pH electrode method, absorption spectroscopy, and flash photolysis to characterize and compare them with the CPD-deleted construct. We found that *Gt*ACR1_full preferentially transported NO_3_^−^, which resulted in an extended lifetime and the large accumulation of the gate-open (NO_3_^−^-conducting) state (discussed below). We also found that the CPD had an inhibitory effect on the intensity of anion transport activity, whereas it enhanced the transport preference for NO_3_^−^ by increasing the specific efficiency for NO_3_^−^ against Cl^−^ (discussed below). To the best of our knowledge, this is the first report that characterizes the full-length ACR expressed in a recombinant system.

### Biological role of the preferential transport of NO_3_^−^ by *Gt*ACR1_full

As shown in Figure 4*B*, the NO_3_^−^ transport activity of *Gt*ACR1_full was about 2 to 4-times larger than that for Cl^−^, Br^−^, I^−^, and SO_4_^2−^, when measured using the pH electrode method (12). Therefore, we speculated that the NO_3_^−^ transport could be the original function of *Gt*ACR1_full in nature and that the CPD contributes to the development of that preference. Conversely, the deletion of the CPD increases the overall anion transport activity but decreases the transport preference.

Konno *et al.* (38) reported that the depletion of nitrogen sources from the culture medium results in the increased expression level of *Gt*ACR1 in native *G. theta* cells. Since the *Gt*ACR1 used in their study was the full-length *Gt*ACR1, taken together with the results of this study (Fig. 4), we suggest that the biological role of *Gt*ACR1_full might be to transport nitrogen in the form of NO_3_^−^ to supplement the nitrogen source. Note that NO_3_^−^ is one of the most stable forms of nitrogen on earth and is used as a nitrogen source by most organisms (39). *G. theta* is a marine cryptophyte alga. In seawater, the concentration ratio between NO_3_^−^ (a few to several tens of mM) and Cl^−^ (500–600 mM) is calculated to be approximately 0.001 to 0.1 (www.resourcewatch.org). As described above, *G. thet*a controls the expression level of *Gt*ACR1 in response to the concentration of extracellular nitrogen source (38). Based on the composition of the culture medium used in the previous study by Konno *et al.* (38), we estimate that the medium contains approximately a half the concentration of Cl^−^ of seawater (approximately 200–300 mM) and up to approximately 4 mM of NO_3_^−^ as a nitrogen source. In addition, it is known that *Proteomonas sulcata*, which is also a marine cryptophyte alga and has ACRs, senses the extracellular NO_3_^−^ (less than 1 mM) and accumulates nitrogen as a form of protein-pigment complex phycoerythrin, which contributes to the light-harvesting function for photosynthesis, even in the presence of about a half the concentration of Cl^−^ of seawater (40, 41). These previous results indicate that NO_3_^−^ is sensed and transported inside the native algal cells even under the low NO_3_^−^/Cl^−^ ratio and strongly negative membrane potential (approximately 100–150 mV) (42, 43), namely under native environment.

If that is the case, then CCRs and ACRs have distinctly different physiological roles. CCRs play a role as a phototaxis sensor triggered by light-dependent photoreceptor current (5). That is, CCRs transport cations, such as H^+^, Na^+^, and Ca^2+^, to induce membrane depolarization in the algal eye spot. On the other hand, ACRs could be responsible for transporting NO_3_^−^ for use as a nutrient source. However, this hypothesis needs to be tested by *in vivo* studies.

### Relationship between photocycle kinetics and anion transport activity

Comparative analysis of photocycle kinetics suggested that in the case of *Gt*ACR1_full, the formation of the M-intermediate, which temporally correlates with the gate-closing (33, 37), was delayed in the presence of NO_3_^−^ compared to *Gt*ACR1_ΔCPD (Fig. 7*E* and τ_2_ in Table 1). In other words, the lifetime of the gate-open (NO_3_^−^-conducting) state per one photocycle is likely to become longer when transporting NO_3_^−^.

The change in photocycle kinetics must change the accumulation of photo-intermediates. Therefore, we discuss the accumulation of the gate-open state to elucidate the relationship between photocycle kinetics and the anion transport activities of *Gt*ACR1_full and *Gt*ACR1_ΔCPD for Cl^−^ and NO_3_^−^, respectively. For this purpose, we focused on accumulation of the L-intermediate because in the case of *Gt*ACR1, that is the only photo-intermediate involved in the gate-open state for transporting anions (33, 37). Using the photocycle kinetics data, we estimated the accumulation of the L-intermediate (see Equation 1 in Supporting information) under continuous light conditions, assuming the experimental condition for anion transport measurements. We estimate that the L-intermediate is responsible for the gate-open state not only in the presence of Cl^−^ (Fig. 6*F*) (33, 37) but also in the presence of NO_3_^−^ (Fig. 7*F*), the latter of which should be experimentally determined in a future study.

**Figure 8:**
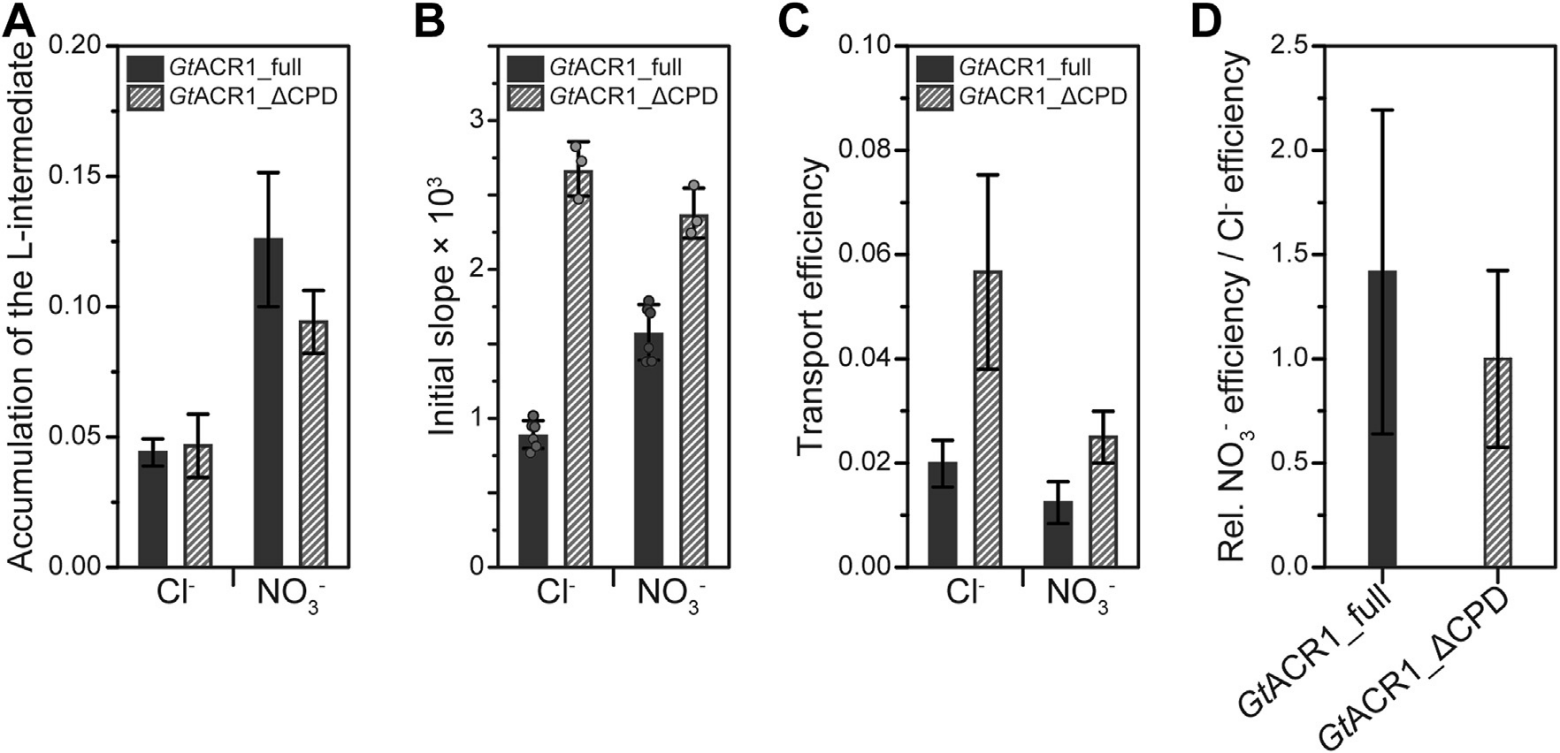
**Relationship between photocycle kinetics and anion transport activity.** The results of *Gt*ACR1_full and *Gt*ACR1_ΔCPD are shown as *solid black* and *striped bars*, respectively. *A*, summary of the accumulation of the gate-open state, L-intermediate, in the presence of Cl^−^ and NO_3_^−^. The details of the calculation are described in Supporting information. Data are reported as means ± S.D. The error bars are originally derived from the S.D. values of the time constants (Table 1), which have been obtained by the global fitting analysis for the flash photolysis data. Data are reported as means ± S.D. For *Gt*ACR1_full, the mean ± S.D. values are 4.41 × 10^−2^ ± 5.23 × 10^−3^ for Cl^−^ and 1.26 × 10^−1^ ± 2.57 × 10^−2^ for NO_3_^−^, respectively. For *Gt*ACR1_ΔCPD, the mean ± S.D. values are 4.66 × 10^−2^ ± 1.21 × 10^−2^ for Cl^−^ and 9.41 × 10^−2^ ± 1.20 × 10^−2^ for NO_3_^−^, respectively. No statistical analysis was performed. *B*, summary of the anion transport activities of *Gt*ACR1_full (*solid black bars*; *n* = 6) and *Gt*ACR1_ΔCPD (*striped bars*; *n* = 3) extracted from Figure 4*B*. Data are reported as means ± S.D. For *Gt*ACR1_full, the mean ± S.D. values are 8.76 × 10^−4^ ± 9.35 × 10^−5^ for Cl^−^ and 1.56 × 10^−3^ ± 1.85 × 10^−4^ for NO_3_^−^, respectively. For *Gt*ACR1_ΔCPD, the mean ± S.D. values are 2.64 × 10^−3^ ± 1.81 × 10^−4^ for Cl^−^ and 2.35 × 10^−3^ ± 1.66 × 10^−4^ for NO_3_^−^, respectively. No statistical analysis was performed. *C*, the anion transport activity (from *panel B*) per accumulation of the L-intermediate (from *panel A*) for Cl^−^ and NO_3_^−^ is summarized, which can be estimated as the transport efficiency. The data represents the mean ± S.D. values calculated using the data shown in *panel A* and *B*. For *Gt*ACR1_full, the mean ± S.D. values are 1.99 × 10^−2^ ± 4.48 × 10^−3^ for Cl^−^ and 1.24 × 10^−2^ ± 4.01 × 10^−3^ for NO_3_^−^, respectively. For *Gt*ACR1_ΔCPD, the mean ± S.D. values are 5.67 × 10^−2^ ± 1.86 × 10^−2^ for Cl^−^ and 2.50 × 10^−2^ ± 4.96 × 10^−3^ for NO_3_^−^, respectively. No statistical analysis was performed. *D*, the specific efficiency for NO_3_^−^ against Cl^−^ calculated from the data shown in *panel C* (transport efficiency for NO_3_^−^ per that for Cl^−^) is presented as a relative value. The data represents the mean ± S.D. values calculated using the mean ± S.D. value shown in *panel A* and *B*. For *Gt*ACR1_full and *Gt*ACR1_ΔCPD, the mean ± S.D. values are 1.42 × 10^−1^ ± 7.77 × 10^−1^ and 1.00 × 10^−1^ ± 4.24 × 10^−1^, respectively. No statistical analysis was performed. ACR, anion channelrhodopsin; CPD, cytoplasmic domain.

Figure 8*A* shows the accumulation of the L-intermediate. For comparison, the results of the transport activities for Cl^−^ and NO_3_^−^ were extracted from Figure 4*B* and are summarized in Figure 8*B*. As a result, in general, the accumulation was larger in the presence of NO_3_^−^ than in the presence of Cl^−^ (Fig. 8*A*). A comparison of each anion shows that in the presence of NO_3_^−^, the accumulation was increased for *Gt*ACR1_full compared with *Gt*ACR1_ΔCPD while in the presence of Cl^−^, the accumulation of *Gt*ACR1_full and *Gt*ACR1_ΔCPD was almost the same. These results reflect that the lifetime of the gate-open state becomes longer when transporting NO_3_^−^, as shown in Figure 7*E*. In addition, these results indicate that the CPD contributes to increasing the accumulation of the gate-open state especially in the presence of NO_3_^−^. We speculate that this results in the preferential transport of NO_3_^−^ by *Gt*ACR1_full (Figs. 4 and 8*B*).

What would happen with the increased accumulation of the gate-open state? From the data shown in Figure 8, *A* and *B*, we calculated the anion transport activity per accumulation of the L-intermediate, which can be estimated as the transport efficiency, as shown in Figure 8*C*. As a result, the transport efficiency was decreased in the case of *Gt*ACR1_full and in the presence of NO_3_^−^. However, the specific efficiency for NO_3_^−^ against Cl^−^ for *Gt*ACR1_full is about 1.4-times larger than that for *Gt*ACR1_ΔCPD (Fig. 8*D*). These results indicate that although the CPD showed an inhibitory effect on anion transport activity (Figs. 4 and 8*B*), it enhances NO_3_^−^ transport activity. In conclusion, the preferential transport activity of NO_3_^−^ by *Gt*ACR1_full is considered to result in the extended lifetime and the large accumulation of the gate-open state and the increase in the specific efficiency for NO_3_^−^ against Cl^−^. To more accurately quantify the accumulation of the gate-open state, the anion transport activity, and efficiency, electrophysiological measurements should be conducted in the future.

What would be a possible molecular mechanism for the preferential NO_3_^−^ transport? How different are the transport mechanisms for NO_3_^−^ and Cl^−^? We discuss these questions in the following sections.

### The CPD may facilitate the influx of NO_3_^−^*via* an interaction with the rhodopsin domain

**Figure 9:**
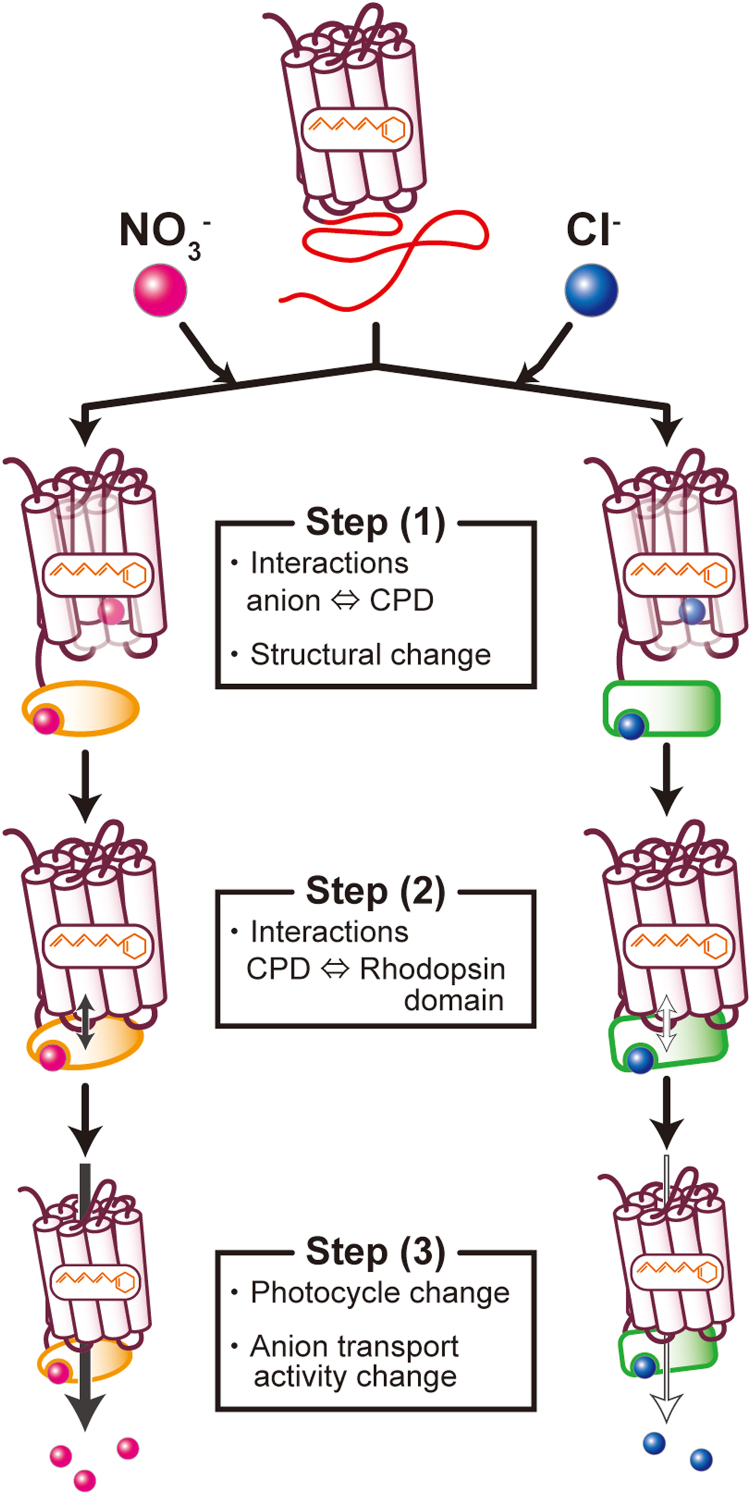
**Possible molecular mechanisms of NO**_**3**_^**−**^**(*left*) and Cl**^**−**^**transport (*right*) by *Gt*ACR1_full.** Step (1): The anions are captured by the CPD. Different folding structures (shown as *orange ovals* for NO_3_^−^ and as *green rectangles* for Cl^−^) were induced depending on the anion species (shown in *pink* for NO_3_^−^ and in *blue* for Cl^−^). Step (2): The CPD interacts with the rhodopsin domain differently (shown as a *black arrow* for NO_3_^−^ and as a *white arrow* for Cl^−^). Step (3): As the result of the different folding structures and the different interactions with the rhodopsin domain, the photocycles for each anion are affected as shown in Figure 6, Figure 8, and therefore the anion transport activities (preference) are also affected as shown in Figure 4. ACR, anion channelrhodopsin; CPD, cytoplasmic domain.

Although analysis of the amino acid sequence did not reveal any known domains in the CPD or in conserved residues (Fig. S2), we show that the anion transport function of *Gt*ACR1 is modulated by the presence of the CPD (Figs. 4 and Figure 6, Figure 8). How does the CPD modulate the function of *Gt*ACR1? The scenario we favor is that (Fig. 9, left-side): Step (1), the CPD captures NO_3_^−^ together with its structural change; step (2), the CPD interacts with the rhodopsin domain; and step (3), as a result, the photocycle is modulated and therefore the CPD further facilitates the influx of NO_3_^−^.

The CPD of *Gt*ACR1 consists of 143 amino acids containing 29 acidic residues (Asp and Glu) and 39 basic residues (Lys and Arg, His is excluded, Fig. S2). As a result, the CPD is positively charged at a neutral pH. Therefore, the CPD should be able to electrostatically interact with NO_3_^−^. At the same time, such interaction may induce and stabilize the folding structure of the CPD. This is a phenomenon called “coupled binding and folding” and is one of the features of intrinsically disordered proteins (27). Moreover, the NO_3_^−^-bound structure of the CPD can further be stabilized by electrostatic interactions between Arg and phosphorylated Ser or Thr in the CPD (Fig. 2*C*, Fig. S8*A*), which is generally known as interactions having a covalent-like stability and contributing to protein–protein interactions (44). At this time, the CPD and NO_3_^−^ need to encounter in the cytoplasmic side of the cell membrane. As shown in Figure 4 and in Govorunova *et al.* (7), *Gt*ACR1 transports NO_3_^−^ even without the CPD, and the CPD does not completely inhibit that transport. Therefore, NO_3_^−^ can penetrate into the cells and we hypothesize that in step (1), the CPD captures NO_3_^−^ inside the algal cells after the initial uptake of NO_3_^−^, after which the captured NO_3_^−^ induces and stabilizes the folding structure of the CPD. We are now conducting structural studies on the CPD to prove that hypothesis.

In the case of *Gt*ACR1_full, as shown in Figure 7*E*, the formation of the M-intermediate at 390 nm was clearly delayed in the presence of NO_3_^−^, meaning that the lifetime of the gate-open (NO_3_^−^-conducting) state was extended. For step (2), we hypothesize that after the CPD captures NO_3_^−^, the NO_3_^−^-bound CPD interacts with the cytoplasmic part of the rhodopsin domain possibly through an electrostatic interaction. In general, the cytoplasmic domains of transmembrane α-helical proteins are positively charged due to the inside-positive rule. In fact, we see several positively charged residues that are located on the cytoplasmic part of the *Gt*ACR1 rhodopsin domain (Fig. S9). On the other hand, the folding structure induced in the CPD after capturing NO_3_^−^ (step (1)) may cause the negatively charged amino acids to cluster on the protein surface. Alternatively, it is possible that the NO_3_^−^-bound CPD interacts with the rhodopsin domain *via* covalent-like electrostatic interactions between Arg on the cytoplasmic surface of the rhodopsin domain and the phosphorylated Ser or Thr in the CPD (Fig. S8*B*) (44).

Haloarchaeal sensory rhodopsins interact with their cognate transducer proteins, which are also transmembrane α-helical proteins, to transmit light sensory signals inside microbial cells to regulate flagellar motility (45, 46). In this case, the interaction prolongs the lifetime of an intermediate of the photocycle that represents the signaling state (47, 48, 49). *Anabaena* sensory rhodopsin also interacts with its transducer protein, which is a soluble protein expressed inside *Anabaena* cells (50). As a result, the photocycle rate becomes 20% faster. The observed modulation of photocycle kinetics in full-length *Gt*ACR1 suggests an intramolecular interaction between the CPD and the rhodopsin domain, in contrast to the sensory rhodopsins described above. We expect that the dissociation constant of the interaction would be smaller than the cases of sensory rhodopsins (tens of micro-molars) (51).

Finally, for step (3), the interactions of the NO_3_^−^-bound CPD and the *Gt*ACR1 rhodopsin domain could further facilitate the NO_3_^−^ influx. As shown in Figures 6 and 7, the photocycle of *Gt*ACR1_full in the presence of NO_3_^−^ is different from that in the presence of Cl^−^ and thus is modulated to achieve the preferential NO_3_^−^ transport activity (discussed below). If this could be experimentally proven *in vivo*, the preferential transport of NO_3_^−^ by *Gt*ACR1_full is under a positive feedback control. In biological systems, it is effective when the production of depleted biological materials is increased simultaneously. Therefore, we speculate that *G. theta* avoids the depletion of nitrogen sources by increasing not only the expression of *Gt*ACR1_full but also the influx of NO_3_^−^ through the protein.

### Dependence of the *Gt*ACR1 photocycle on anions

We showed that the photocycles of *Gt*ACR1_full and *Gt*ACR1_ΔCPD were altered in the presence of Cl^−^ and NO_3_^−^, respectively (Figs. 6 and 7, and Table 1). In the case of *Gt*ACR1_full, one reason for this could result from an interaction between the rhodopsin domain and the anion-bound CPD. In other words, we expect that the CPD can interact with Cl^−^ and NO_3_^−^; however, the resulting structure of the CPD and interactions with the rhodopsin domain are different. Intrinsically disordered proteins are known to interact in a multifaceted manner (27).

In the presence of Cl^−^, the decay of the M-intermediate was significantly delayed for *Gt*ACR1_full compared to *Gt*ACR1_ΔCPD (Fig. 6*E*). This kinetic behavior is clearly different from the case in the presence of NO_3_^−^, in which the formation of the M-intermediate was delayed (Fig. 7*E*). The former delay leads to elongation of the photocycle duration. In addition, the smaller accumulation of the gate-open state for Cl^−^ transport (Fig. 8*A*) resulted in a weaker Cl^−^ transport activity (Figs. 4 and 8*B*) compared to the case of NO_3_^−^. If the disordered CPD also interacts with Cl^−^, the resulting folding structure is speculated to be different from that induced in the presence of NO_3_^−^ (Fig. 9, right-side scheme, step (1)). As a result, the interaction with the *Gt*ACR1 rhodopsin domain may also be changed (step (2)), which induces a delayed decay of the M-intermediate (Fig. 6*E* and step (3)). This also needs to be proven in future research. If this should prove to be true, the function of the CPD would be a precise mechanism that controls anion transport according to physiological needs.

In fact, we have tried to characterize the interactions between the CPD and the *Gt*ACR1 rhodopsin domain by monitoring the change in photocycle kinetics of *Gt*ACR1_ΔCPD before and after mixing with the CPD in the presence of Cl^−^. We prepared the CPD in the *Escherichia coli* expression system and *Gt*ACR1_ΔCPD having a His-tag at the N terminus in the *P. pastoris* expression system (see Experimental procedures in Supporting information, and Fig. S10*A*). However, even after adding a large excess of the CPD (a 10-fold molar ratio) to *Gt*ACR1_ΔCPD, we could not observe a delayed photocycle (Fig. S10*B*) as was the case for *Gt*ACR1_full. This result indicates that the *Gt*ACR1_ΔCPD and the added CPD do not interact with each other. The cause of this might be the loss of phosphorylation of the CPD because the CPD prepared in the *E. coli* system cannot be modified after translation. The phosphorylation of the CPD might be important for its interaction with the rhodopsin domain (Fig. S10*C*).

### Cl^−^ and NO_3_^−^ bind to the rhodopsin domain of *Gt*ACR1 in the initial dark state

Figures 6 and 7 showed that the photocycles of *Gt*ACR1_full and *Gt*ACR1_ΔCPD are altered in the presence of Cl^−^ and NO_3_^−^, respectively. These results prompted us to reconsider the anion-binding ability of the rhodopsin domain of *Gt*ACR1, meaning *Gt*ACR1_ΔCPD, in the initial state. A previous spectroscopic study concluded that *Gt*ACR1_ΔCPD did not bind anions in the initial state because no visible spectral change, that is a color change, was observed when exchanging anions (33), as we also found (Fig. 5*B*). This is a different characteristic from the anion pump HRs, in which spectral (color) changes occur when anions bind in the vicinity of the protonated retinal Schiff base (34, 36, 52, 53, 54, 55). On the other hand, we previously reported that *P. sulcata* ACR1 (*Psu*ACR1), which is closely related to *Gt*ACR1 (identity 36% and similarity 74%, calculated from their amino acid sequences corresponding to the rhodopsin domain), was capable of binding Cl^−^ in the initial state, determined by visible spectral (color) changes similar to HRs (11). In addition to the spectral changes in the visible region, which correspond to the protonated retinal Schiff base, we observed other changes in the near UV region, which correspond to the deprotonated retinal Schiff base. When increasing the Cl^−^ concentration, the absorption band intensity of the deprotonated retinal Schiff base became smaller. This result indicates that the p*K*_a_ of the retinal Schiff base increased together with increasing the Cl^−^ concentration. The simple interpretation for this phenomenon is that the Cl^−^ binds near and electrostatically interacts with the protonated retinal Schiff base, which results in increasing the p*K*_a_ of the retinal Schiff base. This has also been observed in HRs (52, 56, 57, 58, 59). From this background, we conceived the idea that we could observe an increase in the p*K*_a_ of the retinal Schiff base for *Gt*ACR1_ΔCPD in the presence of Cl^−^ and NO_3_^−^, respectively. This is an indirect observation; however, it can indicate possible anion binding to *Gt*ACR1_ΔCPD and interactions between the anions and the protonated retinal Schiff base.

**Figure 10:**
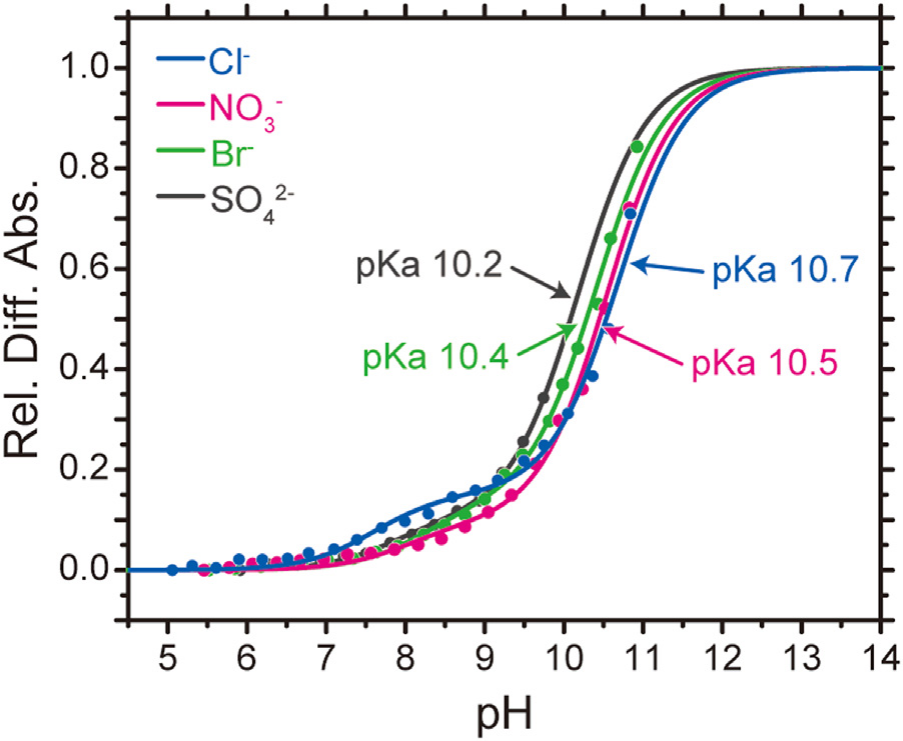
**Anion-dependent shift of the p*K***_**a**_**for the retinal Schiff base.** The pH-titration experiments were performed for *Gt*ACR1-ΔCPD in the presence of 1 M NaCl (*blue*), 1 M NaNO_3_ (*red*), 1 M NaBr (*green*), and 333.3 mM Na_2_SO_4 (*black*)_. The ionic strength was kept at 1 M. *Solid lines* represent the fitting curves analyzed by the Henderson–Hasselbalch equation with two p*K*_a_ values. The p*K*_a_ for the retinal Schiff base is described in the Figure. Currently, the origin of another p*K*_a_ at around 8 is unknown. The UV-visible absolute and difference absorption spectra are summarized in Fig. S10. ACR, anion channelrhodopsin; CPD, cytoplasmic domain.

Thus, we prepared *Gt*ACR1_ΔCPD in the presence of 1 M NaCl, 1 M NaNO_3_, and 1 M NaBr. In addition, we prepared *Gt*ACR1_ΔCPD containing 333.3 mM Na_2_SO_4_ as a comparison to keep the ionic strength at 1 M. Note that *Gt*ACR1_ΔCPD transports little SO_4_^2−^ (Fig. 4) (7, 12). Fig. S11 shows the pH-dependent changes of the absolute and the difference absorption spectra of each sample. When titrating from an acidic to an alkaline pH by adding small amounts of NaOH, the visible absorption bands were commonly decreased with concomitant increases in the near UV absorption bands, indicating the alkaline-induced deprotonation of the retinal Schiff base. In Figure 10, the absorption increase at 370 nm, which corresponds to an increase in the deprotonated state of the retinal Schiff base, was plotted against the pH. Interestingly, the deprotonation of the retinal Schiff base occurred by two steps, indicating that two p*K*_a_s were needed to obtain a good fitting result. The larger p*K*_a_ at around ten can correspond to the retinal Schiff base; however, the origin of the smaller p*K*_a_ at around eight is currently unknown.

In 2021, the Br^−^-bound structure of *Gt*ACR1 was reported (19). The Br^−^-binding site was located at the cytoplasmic side and 16.8 Å away from the Schiff base nitrogen. Therefore, we checked the effect of Br^−^ binding on the p*K*_a_ shift. As a result, the p*K*_a_ was 10.4 in the presence of 1 M Br^−^, which was larger than in the presence of SO_4_^2−^ (10.2) (Fig. 10). This result indicates that the bound Br^−^ indeed affects the p*K*_a_ of the retinal Schiff base even though the binding site is distant from the retinal Schiff base. On the other hand, in the presence of Cl^−^ and NO_3_^−^, the p*K*_a_s were 10.7 and 10.5, respectively. Therefore, from these results, we conclude that *Gt*ACR1_ΔCPD is capable of binding not only Br^−^ but also Cl^−^ and NO_3_^−^ in the initial state as same as *Psu*ACR1 (11). Unfortunately, we were unable to identify the binding sites for Cl^−^ and NO_3_^−^ in this study. Because the photocycle is affected by the anion species (Figs. 6 and 7), we assume that the anion binding in the initial state is of some functional benefit for *Gt*ACR1, for example, anion transport preference/selectivity.

## Conclusion

We suggest that the original function of full-length *Gt*ACR1, which has been overlooked in previous studies, is to preferentially transport NO_3_^−^ in nature. The preferential NO_3_^−^ transport of *Gt*ACR1_full was resulted from the extended lifetime and the large accumulation of the gate-open (NO_3_^−^-conducting) state. These results also revealed that the CPD has an inhibitory effect on the intensity of anion transport activity, whereas it contributes to the development of transport preference for NO_3_^−^ by increasing the specific efficiency for NO_3_^−^ against Cl^−^. Although some hypothetical mechanisms need to be elucidated in the future, such as the mechanism of anion selection by the intrinsically disordered CPD, the role of phosphorylation of the protein, and the positive feedback control of NO_3_^−^ transport *in vivo*, we have certainly learned some new facts thanks to the successful preparation of full-length *Gt*ACR1 as a recombinant protein. We believe that our study provides important new experimental data and insights into life activities from a molecular perspective.

## Experimental procedures

### DNA construction of *Gt*ACR1

DNA and amino acid sequences of *Gt*ACR1 were taken from the JGI PhycoCosm genomic database (Protein ID: 111593) (7). *Gt*ACR1_full and *Gt*ACR1_ΔCPD are composed of 438 and 295 amino acids, respectively. An eight-histidine-tag was attached to the C terminus of each protein sequence. *Gt*ACR1 genes with codon optimization for expression in *P. pastoris* were purchased from GENEWIZ (South Plainfield). The procedures for constructing the pPICZ B vector (Thermo Fisher Scientific) for *P. pastoris* were the same as our previous reports (11, 12) and are described in Supporting information. The authenticity of all DNA sequences was confirmed by dideoxy sequencing.

### Protein expression and purification

The methylotrophic yeast *P. pastoris* SMD1168H strain (Thermo Fisher Scientific) was used as the protein expression host. The procedures for transformation of the yeast, protein expression, and protein purification were the same as our previous reports (11, 12) and are described in Supporting information.

### Anion transport activity measurement using the pH electrode method and data analysis

The procedures were almost the same as our previous report (12). To measure anion transport activity, the *P. pastoris* SMD1168H cell density was adjusted by monitoring the optical density at 660 nm to 10 on average using a UV-1800 spectrophotometer (Shimadzu Corp). For activation, green (peak wavelength is 530 nm) LED light (47 mW/cm^2^ on average, ORION, Ophir Optronics Solutions Ltd) was illuminated for 2 min. To reduce large artifacts on the pH electrode from such a strong light, the internal KCl solution was replaced with 3.3 M KCl dissolved in India ink (60). The anion transport activity was determined by the initial slope of the first 10 s after LED light illumination for time-dependent pH changes. The measurements for *Gt*ACR1_full and *Gt*ACR1_ΔCPD were repeated six and three times, respectively. To quantify and correct differences in protein expression levels, SDS-PAGE and Western blotting were performed using the same procedures reported previously (12). Details are described in Supporting information. Three independent measurements were averaged. For statistical analysis, unpaired t-tests were performed using GraphPad Prism 9 software, https://www.graphpad.com.

### Spectroscopic measurements

Static UV-visible absorption spectra were recorded at room temperature using a UV-1800 spectrophotometer (Shimadzu Corp). Flash photolysis measurements for time-dependent absorption changes were performed using a homemade computer-controlled apparatus (61). The temperature was maintained at 20 °C. For the measurements, data for time-dependent absorption changes at 350 to 700 nm every 10 nm were obtained. The number of data acquisitions was 200 for 350 to 400 nm and 50 for 410 to 700 nm. Data were analyzed by the sequential model as reported previously (62). The photocycle model was constructed by reference to Sineshchekov *et al.* (33) and Dreier *et al.* (63). The experimental details are described in Supporting information. The buffer conditions for UV-visible spectroscopy and flash photolysis were 10 mM 2-[4-(2-Hydroxyethyl)-1-piperazinyl]ethanesulfonic acid (pH 7.5), 1 M salt (NaCl, NaBr, NaI, NaNO_3_, Na_2_SO_4_), and 0.05% DDM (Dojindo).

For pH titration experiments, *Gt*ACR1_ΔCPD was suspended in a mix of six buffers (0.89 mM citrate, 0.89 mM MES, 1.1 mM TES, 0.78 mM TAPS, 1.1 mM CHES, and 0.33 mM CAPS) containing 0.05% DDM and salts (1 M NaCl, 1 M NaNO_3_, 1 M NaBr, or 333.3 mM Na_2_SO_4_). The initial pH was around 5. The ionic strength was kept at 1 M. A small amount of 0.1 M NaOH solution was added to the sample solution. Difference UV-visible absorption spectra were calculated by subtracting the spectrum at the initial pH from the others. The difference absorbance at 370 nm, Δ*Abs*_370_, was plotted against the measured pH. The difference absorbance is presented as a relative value, which was calculated by taking into account the percentage of *Gt*ACR1_ΔCPD deprotonated at an alkaline pH. The data were fitted with the Henderson–Hasselbalch equation with two p*K*_a_ values:

$${\Delta Abs}_{370}=\frac{A}{1+10^{\left(pK_{a,1}-pH\right)}}+\frac{1-A}{1+10^{\left(pK_{a,2}-pH\right)}}$$

where A represents the amplitude. To obtain optimal fitting results, we analyzed the data by the two-p*K*_a_ model (p*K*_a,1_ < p*K*_a,2_). p*K*_a,2_ corresponds to the p*K*_a_ of retinal Schiff base. Unfortunately, the origin of the p*K*_a,1_ at around eight is currently unknown.

## Data availability

All the data supporting the findings in this research are available within the article and the Supporting information.

## Supporting information

This article contains Supporting information (7, 11, 12, 13, 14, 15, 32, 44, 60, 61, 62, 63, 64).

## Conflict of interest

The authors declare that they have no conflicts of interest with the contents of this article.
