# Supplementary material for: The preferential transport of NO3− by full-length Guillardia theta anion channelrhodopsin 1 is enhanced by its extended cytoplasmic domain
Source: J Biol Chem. 2023 Sep 29;299(11):105305. doi: 10.1016/j.jbc.2023.105305 (PMC10637977; doi:10.1016/j.jbc.2023.105305)
Supplement: Supporting Information [file mmc1.docx]

**Supporting information for**

**The preferential transport of NO_3_^-^ by full-length *Guillardia theta* anion channelrhodopsin 1 is enhanced by its extended cytoplasmic domain**

**Yuya Ohki^1^, Tsukasa Shinone^2^, Sayo Inoko^2^, Miu Sudo^2^, Makoto Demura^3^, Takashi Kikukawa^3^, Takashi Tsukamoto^3,*^**

^1^ Division of Soft Matter, Graduate School of Life Science, Hokkaido University, Sapporo 060-0810, Japan.

^2^ Division of Macromolecular Functions, Department of Biological Science, School of Science, Hokkaido University, Sapporo 060-0810, Japan.

^3^ Faculty of Advanced Life Science, Hokkaido University, Sapporo 060-0810, Japan.

^*^Corresponding author: Takashi Tsukamoto

Email: t-tak@sci.hokudai.ac.jp

**Contents:**

1. **Experimental procedures**

- *DNA construction of GtACR1*
- *DNA constructions of CPD and GtACR1_ΔCPD with a N-terminal His-tag*
- *Protein expression and purification of GtACR1 proteins*
- *Protein expression and purification of CPD*
- *Anion transport activity measurement using the pH electrode method and data analysis*
- *Spectroscopic measurements*
- *Estimation of the effective accumulation of the gate-open state based on flash photolysis*
- *Flash photolysis measurement to demonstrate interactions between the GtACR1 rhodopsin domain and the CPD*

1. **Supporting Figures**

- *S1. Amino acid sequence alignment of the rhodopsin domain of various ACRs*
- *S2. Amino acid sequence alignment of the CPD of various ACRs*
- *S3. Spectral separation of the P_i_ states (i = 0 – 4) of GtACR1_full in the presence of Cl^-^*
- *S4. Spectral separation of the P_i_ states (i = 0 – 4) of GtACR1_ΔCPD in the presence of Cl^-^*
- *S5. Spectral separation of the P_i_ states (i = 0 – 4) of GtACR1_full in the presence of NO_3_^-^*
- *S6. Spectral separation of the P_i_ states (i = 0 – 4) of GtACR1_ΔCPD in the presence of NO_3_^-^*
- *S7. Illustrations of covalent-like electrostatic interactions between Arg and phosphorylated Ser/Thr residues*
- *S8. The structure of GtACR1 modeled by AlphaFold2*
- *S9. Demonstration of the interaction between the CPD and the rhodopsin domain of GtACR1_ΔCPD using flash photolysis*
- *S10. pH titration of the UV-visible absorption spectra of GtACR1_ΔCPD*

1. **References**
2. **Experimental procedures**

**DNA construction of *Gt*ACR1.**

DNA and amino acid sequences of *Gt*ACR1 were taken from the JGI PhycoCosm genomic database (Protein ID: 111593) (1). The full-length *Gt*ACR1 (*Gt*ACR1_full) and *Gt*ACR1 without the cytoplasmic domain (CPD) (*Gt*ACR1_ΔCPD) are composed of 438 and 295 amino acids, respectively. An eight-histidine-tag was attached to the C-terminus of each protein sequence. *Gt*ACR1 genes with codon optimization for expression in *Pichia pastoris* were purchased from GENEWIZ (South Plainfield, NJ, U.S.A.).

The following procedures were the same as our previous reports (2, 3). The codon-optimized *Gt*ACR1 genes were amplified by PCR using KOD -Plus- Neo DNA polymerase (TOYOBO Co., Ltd., Osaka, Japan). The sequences of PCR primers included EcoRI and NotI sites at the 5’ and 3’ termini. The amplified *Gt*ACR1 genes and the pPICZ B vector (Thermo Fisher Scientific, Waltham, MA, USA) were digested using EcoRI and NotI restriction enzymes (FastDigest^TM^, ThermoFisher Scientific). After purification, the *Gt*ACR1 genes were incorporated into the pPICZ B vector using a DNA Ligation Kit <Mighty Mix> (Takara Bio Inc., Shiga, Japan). The authenticity of each DNA sequence was confirmed by dideoxy sequencing. For transformation of the methylotrophic yeast *Pichia pastoris* SMD1168H strain (ThermoFisher Scientific), 50 – 60 μg of each DNA was prepared using a QIAGEN Plasmid Midi Kit (QIAGEN, Venlo, Netherlands) and was linearized using PmeI restriction enzyme (New England BioLabs, Inc., Ipswich, MA, U.S.A.).

**DNA constructions of CPD and *Gt*ACR1_ΔCPD with a N-terminal His-tag.**

To examine the interaction between the rhodopsin domain and the CPD of *Gt*ACR1, we prepared *Gt*ACR1_ΔCPD with a N-terminal six-histidine-tag using the *Pichia pastoris* expression system and CPD with a C-terminal six-histidine-tag using the *Escherichia coli* expression system. Genes with codon optimization for each expression system were purchased from GENEWIZ.

When constructing the *Gt*ACR1_ΔCPD gene with a N-terminal six-histidine-tag, nucleic acids encoding the amino acid sequence of MHHHHHH were added to the N-terminus of *Gt*ACR1_ΔCPD. The procedures for cloning this gene into the pPICZ B vector were the same as described above.

The codon-optimized CPD gene was amplified by PCR using KOD -Plus- Neo DNA polymerase (TOYOBO Co., Ltd., Osaka, Japan). The sequences of the PCR primers included NdeI and XhoI sites at the 5’ and 3’ termini. The amplified CPD gene and the pET22b vector were digested using NdeI and XhoI restriction enzymes (FastDigest^TM^, ThermoFisher Scientific). After purification, the CPD gene was incorporated into the pET22b vector using a DNA Ligation Kit <Mighty Mix> (Takara Bio Inc., Shiga, Japan). The authenticity of each DNA sequence was confirmed by dideoxy sequencing.

**Protein expression and purification of *Gt*ACR1 proteins.**

The following procedures were the same as our previous reports (2, 3). The methylotrophic yeast *Pichia pastoris* SMD1168H strain was used as the protein expression host. According to the manufacturer’s instructions (EasySelect^TM^ *Pichia* Expression Kit, Cat. no. K1740-01, ThermoFisher Scientific), *P. pastoris* competent cells were prepared after which the linearized DNAs of pPICZ B_*Gt*ACR1_full, pPICZ B_*Gt*ACR1_ΔCPD (C-terminal His_8_-tag) and pPICZ B_*Gt*ACR1_ΔCPD (N-terminal His_6_-tag) were introduced into the cells by electroporation using a MicroPulser electroporator (Bio-Rad Laboratories, Inc., Hercules, CA, U.S.A.). After seeding the cells on YPDS agar plates (1% (w/v) yeast extract, 2% (w/v) peptone, 2% (w/v) glucose, 1 M sorbitol, 2% (w/v) agar) containing 100 – 2000 μg/mL Zeocin^TM^ (ThermoFisher Scientific)), the protein expression levels of *Gt*ACR1_full and *Gt*ACR1_ΔCPD were examined using several colonies of *P. pastoris* cells, and colonies with the highest expression levels were used in the experiment. The expression level was judged from the color of the cells.

Pre-culture was performed using BMGY medium (1% (w/v) yeast extract, 2% (w/v) peptone, 100 mM potassium phosphate buffer (pH 6.0), 1.34% (w/v) yeast nitrogen base with ammonium sulfate, 4 × 10^-5^% (w/v) biotin, 1% (v/v) glycerol) for 1 day at 30^o^C in a shaker incubator (BR-22FP, TAITEC Corp., Saitama, Japan). The main culture was then performed using BMMY medium (1% (w/v) yeast extract, 2% (w/v) peptone, 100 mM potassium phosphate buffer (pH 6.0), 1.34% (w/v) yeast nitrogen base with ammonium sulfate, 4 × 10^-5^% (w/v) biotin, 2% (v/v) methanol) containing 10 μM all-*trans*-retinal (Sigma-Aldrich, St. Louis, MO, U.S.A.) for 1 day at 30^o^C in a shaker incubator (BR-43FH, TAITEC Corp.).

The cultured *P. pastoris* cells were collected by centrifugation, resuspended in 50 mM Tris-HCl buffer (pH 8.0) containing 150 mM NaCl, and then disrupted using a French pressure cell (4^o^C, 100 MPa, 10-times; Ohtake, Tokyo, Japan) or a BeadBeater system with an ice-water jacket (glass beads with 0.5 mm diameter; 3 min, 5-times; BioSpec Products, Bartlesville, OK, USA). The cell suspension was centrifuged at 6,000 rpm for 5 min at 4^o^C (himac CF16RN equipped with a T9A31 rotor; Hitachi Koki Co., Ltd., Tokyo, Japan) and the supernatant containing the membrane fraction was collected. The membrane fraction was collected by ultracentrifugation (4^o^C, 45,000 rpm, 1.5 hr; CP90NX equipped with a P70AT rotor; Hitachi Koki Co., Ltd.), then homogenized and finally solubilized using 1.5% (w/v) *n*-dodecyl-β-D-maltoside (DDM; Dojindo Laboratories, Kumamoto, Japan) at room temperature for 1 hr. After ultracentrifugation again, the supernatant was applied to a HisTrap FF column (5 mL, Cytiva, Tokyo, Japan), which had been washed and equilibrated using 50 mM Tris-HCl buffer (pH 8.0) containing 150 mM NaCl. After the protein had attached to the column, 40 mL wash buffer (50 mM Tris-HCl (pH 8.0), 1 M NaCl, 0.05% (w/v) DDM, 70 mM imidazole) was applied to the column. Finally, the *Gt*ACR1_full and *Gt*ACR1_ΔCPD proteins were obtained by applying an elution buffer (50 mM Tris-HCl (pH 7.0), 1 M NaCl, 0.1%(w/v) DDM, 1 M imidazole) to the column.

**Protein expression and purification of CPD.**

The *Escherichia coli* BL21(DE3) strain was used as the protein expression host. The pET22b_CPD was introduced into *E. coli* competent cells, which were prepared by the CaCl_2_ method and by the standard heat shock method (42^o^C, 45 sec). After seeding the cells on LB plates (1% (w/v) tryptone, 0.5% (w/v) yeast extract, 1% (w/v) NaCl, 2% (w/v) agar) containing 50 μg/mL ampicillin, some colonies were picked up for cell culture.

Pre-culture was performed using 50 mL LB medium containing 50 μg/mL ampicillin overnight at 37^o^C in a shaker incubator (BR-22FP, TAITEC Corp.). The main culture was also performed using 1 L LB medium containing 50 μg/mL ampicillin at 37^o^C in a shaker incubator (BR-43FH, TAITEC Corp.). When the optical density at 660 nm of the *E. coli* cell culture reached around 0.7, 1 mM isopropyl-β-D(-)-thiogalactopyranoside (IPTG) was added to the culture medium to induce protein expression. *E. coli* cells were then collected by centrifugation, resuspended in 50 mM Tris-HCl buffer (pH 8.0) containing 150 mM NaCl, and then disrupted using an ultrasonic homogenizer UD-211 (output 5, duty 50, 10 min; TOMY Seiko Co., Ltd., Tokyo, Japan). The cell suspension was centrifuged at 6,000 rpm for 5 min at 4^o^C (himac CF16RN equipped with a T9A31 rotor; Hitachi Koki Co., Ltd.) and the supernatant was collected. The membrane fraction was removed by ultracentrifugation (4^o^C, 45,000 rpm, 1.5 hr; CP90NX equipped with a P70AT rotor; Hitachi Koki Co., Ltd.). The supernatant was then applied to a HisTrap FF column (5 mL, Cytiva), which had been washed and equilibrated using 50 mM Tris-HCl buffer (pH 8.0) containing 150 mM NaCl. After the protein attached to the column, 40 mL wash buffer (50 mM Tris-HCl (pH 8.0), 1 M NaCl, 0.05% (w/v) DDM, 70 mM imidazole) was applied to the column. Finally, the CPD protein was obtained by applying an elution buffer (50 mM Tris-HCl (pH 7.0), 1 M NaCl, 0.1% (w/v) DDM, 1 M imidazole) to the column. The wash and elution steps were performed using a BioLogic DuoFlow chromatography system (Bio-Rad Laboratories, Inc.) by monitoring the absorbance at 280 nm. Finally, SDS-PAGE was performed to check the purity of the sample.

**Anion transport activity measurement using the pH electrode method and data analysis.**

To measure anion transport activity, we used the pH electrode method as reported previously (3). *Gt*ACR1-expressing *P. pastoris* SMD1168H cells were washed with 300 mM salt solution (NaF, NaCl, NaBr, NaI, NaNO_3_, Na_2_SO_4_, sodium aspartate; all from Fuji Film Wako Chemical Industries, Co., Ltd., Osaka, Japan) 4 times by centrifugation (4^o^C, 3,000 rpm; himac CF16RN equipped with a T9A31 rotor; Hitachi Koki). The cells were resuspended in the same solution used for washing, and the cell density was adjusted to 10 on average by monitoring 660 nm light scattering using a UV-1800 spectrophotometer (Shimadzu Corp., Kyoto, Japan). Anion transport activity was measured using a pH meter LAQUA F-72 equipped with a standard ToupH pH electrode (HORIBA, Ltd., Kyoto, Japan). For activation, green (peak wavelength is 530 nm) LED light (47 mW/cm^2^ in average, ORION, Ophir Optronics Solutions Ltd., Jerusalem, Israel) was illuminated for 2 min. To reduce large artifacts on the pH electrode from such a strong light, the internal KCl solution was replaced with 3.3 M KCl dissolved in India ink (4). The anion transport activity was determined by the initial slope of the first 10 s after the LED light illumination to determine time-dependent pH changes. Six and three independent measurements were averaged for *Gt*ACR1_full and *Gt*ACR1_ΔCPD, respectively. For statistical analysis, unpaired t-tests were performed using GraphPad Prism 9 software (Boston, MA, USA).

To quantify and correct the different protein expression levels, SDS-PAGE and Western blotting were performed using the procedures reported previously (3). SDS-PAGE with 12% (v/v) polyacrylamide gels and Western blotting using an anti-His tag mono-antibody conjugated with horseradish peroxidase (anti-His-tag mAb-HRP-DirecT, MBL Co., Ltd., Nagoya, Japan) were performed using standard protocols. The *P. pastoris* cell samples were prepared using the same procedure published by Kushnirov et al. (5). One mL of each cell suspension used to measure the anion transport activity was collected by centrifugation to make cell pellets. The wet weight of the cell pellets was 10 mg on average. The cell pellets were suspended in 1 mL 0.1 M NaOH and then incubated for 5 min at room temperature. After another centrifugation to remove the supernatant, the cell pellets were resuspended in 250 μL SDS-PAGE sample buffer (60 mM Tris–HCl (pH 6.8), 2% (w/v) SDS, 5% (v/v) glycerol, 4% (v/v) β-mercaptoethanol and 0.0025% (w/v) bromophenol blue) and then boiled at 95°C for 5 min. After another centrifugation, 6 μL of each supernatant was diluted by one-sixth and was loaded on the gel. To estimate the total amount of *Gt*ACR1_full and *Gt*ACR1_ΔCPD expressed in the *P. pastoris* cells, the band intensities were analyzed using ImageJ software (6) and three independent measurements were averaged. We corrected the data for the anion transport activities (the time-dependent pH changes) of *Gt*ACR1_full by multiplying by 100/24, assuming that the *Gt*ACR1_full has the same expression level as the *Gt*ACR1_ΔCPD in *P. pastoris* cells. For statistical analysis, an unpaired t-test was performed using GraphPad Prism 9 software.

**Spectroscopic measurements.**

For spectroscopic measurements, buffer exchange was performed for the purified *Gt*ACR1_full and *Gt*ACR1_ΔCPD samples using Amicon Ultra centrifugal ultrafiltration filters (50,000 MWCO; 4^o^C, 4,000 × *g*; Merck-Millipore, Burlington, MA, U.S.A.) and a PD-10 desalting column (Cytiva). The buffer condition for UV-visible spectroscopy and flash photolysis was 10 mM 2-[4-(2-Hydroxyethyl)-1-piperazinyl]ethanesulfonic acid (HEPES, Dojindo) (pH 7.5), 1 M salts (NaCl, NaBr, NaI, NaNO_3_, Na_2_SO_4_) and 0.05% DDM (Dojindo).

UV-visible absorption spectra were recorded at room temperature using a UV-1800 spectrophotometer (Shimadzu Corp.). For spectral titration, *Gt*ACR1_ΔCPD was resuspended in a mix of 6 buffers (0.89 mM citrate, 0.89 mM MES, 1.1 mM TES, 0.78 mM TAPS, 1.1 mM CHES and 0.33 mM CAPS) containing 0.05% DDM and salts (1 M NaCl, 1 M NaNO_3_, 1 M NaBr or 333.3 mM Na_2_SO_4_). The initial pH was around 5 and the ionic strength was kept at 1 M. A small amount of 0.1 M NaOH solution was added to the sample solution. Therefore, the change of protein concentration was ignored. Difference UV-visible absorption spectra were calculated by subtracting the spectrum at initial pH from the others. The difference absorbance at 370 nm was plotted against the measured pH. The difference absorbance was presented as a relative value, calculated by taking into account the percentage of *Gt*ACR1_ΔCPD deprotonated at alkaline pH. The data were fitted with the Henderson-Hasselbalch equation with two p*K*_a_ values:

$$\Delta Abs@370 nm=\frac{A}{1+{10}^{(pK_{a,1}-pH)}}+\frac{1-A}{1+{10}^{(pK_{a,2}-pH)}}$$

where $A$ represents the amplitude. To obtain optimal fitting results, we analyzed the data by the two p*K*_a_ model (p*K*_a,1_ < p*K*_a,2_), where p*K*_a,2_ corresponds to the p*K*_a_ of retinal Schiff base. Unfortunately, the origin of the p*K*_a,1_ is currently unknown.

Flash photolysis measurements for time-dependent absorption spectroscopy were performed using a homemade computer-controlled apparatus equipped with a Nd-YAG laser (532 nm, 6 ns, 0.4 mJ/pulse, Continuum, Milpitas, CA, U.S.A.) at 20^o^C (7). For flash photolysis measurements, data for time-dependent absorption changes at 350 – 700 nm every 10 nm were obtained. The number of data acquisitions was 200 for 350 – 400 nm and 50 for 410 – 700 nm. Data were analyzed by the sequential model as reported previously (8, 9):

𝑃_0_→𝑃_1_→𝑃_2_→𝑃_3_→𝑃_4_→𝑃_0_

where *P*_0_ and *P*_1_ – *P*_4_ represent the initial state and the 1^st^ – 4^th^ kinetically defined states, respectively. All data for the time-dependent absorption changes were simultaneously fitted with a sum of 4 exponential decay functions in this study. The number of exponents was determined by the reductions in the standard deviation of the residuals. In the *P*_1_ – *P*_4_ states, physically defined photo-intermediates, such as K, L, M and N/O, were populated at equilibrium. The photocycle model was constructed by referring to Sineshchekov et al. (10) and Dreier et al. (11). To determine the absolute spectra of each *P*_i_ state, the pure retinal spectrum was extracted by spectral decomposition as described previously (8, 9) and was used as the spectrum of *P*_0_. Finally, the absolute spectra of *P*_i_ states were obtained by adding the spectrum of *P*_0_ to the absorption differences.

**Estimation of the effective accumulation of the gate-open state based on flash photolysis.**

To elucidate the relationship between the photocycle kinetics and the anion transport activities of *Gt*ACR1_full and *Gt*ACR1_ΔCPD for Cl^-^ and NO_3_^-^, respectively, we estimated the effective accumulation of the gate-open state under continuous light, which is equal to the experimental conditions of the anion transport measurements. In this case, the accumulated amount of the photo-intermediates per unit time can be approximated to be constant. Therefore, the steady state approximation can be made. The amounts of the kinetically-defined intermediate states, *P*_0_ – *P*_4_, can be calculated from the following Scheme 1:


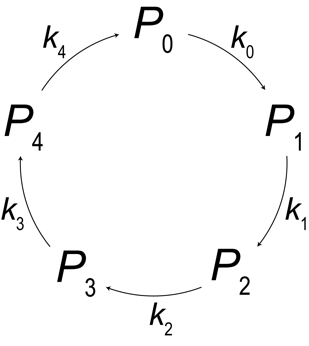


(Scheme 1)

where *k*_0_ – *k*_4_ represent the rate constants of each *P*_0_ – *P*_4_ state. The time-dependent changes of the accumulation (concentration) in each state, $\left[ P_{i} \right]$ (*i* = 0, 1, 2, 3, 4), can be expressed as follows:

$$\frac{d\left[ P_{1} \right]}{dt}=k_{0}\left[ P_{0} \right]-k_{1}\left[ P_{1} \right]$$

$$\frac{d\left[ P_{2} \right]}{dt}=k_{1}\left[ P_{1} \right]-k_{2}\left[ P_{2} \right]$$

$$\frac{d\left[ P_{3} \right]}{dt}=k_{2}\left[ P_{2} \right]-k_{3}\left[ P_{3} \right]$$

$$\frac{d\left[ P_{4} \right]}{dt}=k_{3}\left[ P_{3} \right]-k_{4}\left[ P_{4} \right]$$

$$\frac{d\left[ P_{0} \right]}{dt}=k_{4}\left[ P_{4} \right]-k_{0}\left[ P_{0} \right]$$

Due to the steady state approximation,

$$\frac{d\left[ P_{0} \right]}{dt}=\frac{d\left[ P_{1} \right]}{dt}=\frac{d\left[ P_{2} \right]}{dt}=\frac{d\left[ P_{3} \right]}{dt}=\frac{d\left[ P_{4} \right]}{dt}=0$$

In addition, the total amounts of *P*_0_ – *P*_4_ are constant and thus can be considered to be 1:

$$\left[ P_{0} \right]+\left[ P_{1} \right]+\left[ P_{2} \right]+\left[ P_{3} \right]+\left[ P_{4} \right]=const.=1$$

From the above, solving the differential equation, the accumulation of each state can be described as:

$$\left[ P_{i} \right]=\frac{\tau_{i}}{\tau_{0}+\tau_{1}+\tau_{2}+\tau_{3}+\tau_{4}}$$

where *τ*_i_ represents the time constant of the *i*-th (*i* = 0, 1, 2, 3, 4) states. The time constant *τ*_i_ is the inverse of the rate constant *k*_i_.

From the results of flash photolysis analysis, the gate-open state L-intermediate appears in the *P*_1_, *P*_2_ and *P*_3_ states (Figures 6C, 6D, 7C, and 7D, Supporting Figures S4 – S7). When the molar fractions of the L-intermediate in the *P*_1_, *P*_2_ and *P*_3_ states are described as $f_{P_{1,L}}$, $f_{P_{2,L}}$, and $f_{P_{3,L}}$, the ratio of L in each state, *P*_1,L_, *P*_2,L_ and *P*_3,L_ can be expressed as:

$$\left[ P_{1,L} \right]=\frac{\tau_{1}}{\tau_{0}+\tau_{1}+\tau_{2}+\tau_{3}+\tau_{4}}\times f_{P_{1,L}}$$

$$\left[ P_{2,L} \right]=\frac{\tau_{2}}{\tau_{0}+\tau_{1}+\tau_{2}+\tau_{3}+\tau_{4}}\times f_{P_{2,L}}$$

$$\left[ P_{3,L} \right]=\frac{\tau_{3}}{\tau_{0}+\tau_{1}+\tau_{2}+\tau_{3}+\tau_{4}}\times f_{P_{3,L}}$$

The absorption of the pure L-intermediate in each state, ${Abs}_{P_{1,L}}$, ${Abs}_{P_{2,L}}$, and ${Abs}_{P_{3,L}}$, which are experimentally defined (Supporting Figures S4 – S7), can be expressed as follows:

$${Abs}_{P_{1,L}}=\varepsilon_{P_{1,L}}\times f_{P_{1,L}}$$

$${Abs}_{P_{2,L}}=\varepsilon_{P_{2,L}}\times f_{P_{2,L}}$$

$${Abs}_{P_{3,L}}=\varepsilon_{P_{3,L}}\times f_{P_{3,L}}$$

where $\varepsilon_{P_{1,L}}$, $\varepsilon_{P_{2,L}}$ and $\varepsilon_{P_{3,L}}$ represent the molar extinction coefficients of the L-intermediate in the *P*_1_, *P*_2_, and *P*_3_ states, respectively. Here, if we assume that the extinction coefficients are independent from the length of the protein sequence (*Gt*ACR1_full and *Gt*ACR1_ΔCPD) and the anion species (Cl^-^ and NO_3_^-^), then:

$$\varepsilon_{P_{1,L}}=\varepsilon_{P_{2,L}}=\varepsilon_{P_{3,L}}$$

Therefore, the molar fractions are considered to be the same as the absorption of the pure L-intermediate:

$$f_{P_{1,L}}\approx{Abs}_{P_{1,L}}$$

$$f_{P_{2,L}}\approx{Abs}_{P_{2,L}}$$

$$f_{P_{3,L}}\approx{Abs}_{P_{3,L}}$$

As a result,

$$\left[ P_{1,L} \right]=\frac{\tau_{1}}{\tau_{0}+\tau_{1}+\tau_{2}+\tau_{3}+\tau_{4}}\times{Abs}_{P_{1,L}}$$

$$\left[ P_{2,L} \right]=\frac{\tau_{2}}{\tau_{0}+\tau_{1}+\tau_{2}+\tau_{3}+\tau_{4}}\times{Abs}_{P_{2,L}}$$

$$\left[ P_{3,L} \right]=\frac{\tau_{3}}{\tau_{0}+\tau_{1}+\tau_{2}+\tau_{3}+\tau_{4}}\times{Abs}_{P_{3,L}}$$

Therefore, the effective accumulation of the gate-open state, which is equal to the accumulation of the L-intermediate, can be expressed as the following summation:

$$\left[ P_{1,L} \right]+\left[ P_{2,L} \right]+\left[ P_{3,L} \right]=\frac{\tau_{1}\times{Abs}_{P_{1,L}}+\tau_{2}\times{Abs}_{P_{2,L}}+\tau_{3}\times{Abs}_{P_{3,L}}}{\tau_{0}+\tau_{1}+\tau_{2}+\tau_{3}+\tau_{4}}$$

Again, the absorption of the pure L-intermediate (${Abs}_{P_{1,L}}$, ${Abs}_{P_{2,L}}$, ${Abs}_{P_{3,L}}$) and the time constants ($\tau_{1},\tau_{2},\tau_{3},\tau_{4}$) have been determined in the process of flash photolysis analysis. These values are summarized in Table 1.

Then, we have to consider $\tau_{0}$. The number of *Gt*ACR1 proteins to be excited by laser pulse, $N$, is proportional to the light intensity, $I$:

$$N=\beta\times c\times I$$

where $\beta$ is a proportional constant and $c$ is a molar concentration. The excitation ratio, $F_{c}$, is described as $N$ divided by $c$:

$$F_{c}=\frac{N}{c}=\beta\times I$$

Therefore,

$$\beta=\frac{F_{c}}{I}$$

From our experimental conditions for flash photolysis, we set $I$ to be 0.4 mJ/pulse (see “*Spectroscopic measurements*” in the Experimental procedures section). This is equal to 0.4 mW when assuming that the total photon count remains the same and takes 1 sec to fly toward the sample.

On the other hand, the number of *Gt*ACR1 proteins to be excited by continuous light can be expressed as follows:

$$N=k_{0}\left[ P_{0} \right]=\beta\times c\times I'$$

where $I'$ is the continuous light intensity. We set this to be 47 mW in this study (see “*Anion transport activity measurement using a pH electrode method and data analysis*” in the Experimental procedures section). Because $\left[ P_{0} \right]=c$, the $k_{0}$ can be expressed as:

$$k_{0}=\beta\times I'$$

The $\beta$ should be the same value even when *Gt*ACR1 proteins are embedded in the *P. pastoris* membrane or are solubilized in detergent solution. Therefore,

$$\beta=\frac{F_{c}}{I}=\frac{k_{o}}{I'}$$

$$\tau_{o}=\frac{1}{k_{o}}=\frac{I}{I'}\times\frac{1}{F_{c}}=\frac{0.4}{47}\times\frac{1}{F_{c}}=\frac{1}{117.5\times F_{c}}$$

In the process of flash photolysis analysis, we estimated $F_{c}$ values in each experimental condition as summarized in Table 1. Finally, the effective population of the gate-open state can be expressed as the following Equation 1:

$$\left[ P_{1,L} \right]+\left[ P_{2,L} \right]+\left[ P_{3,L} \right]=\frac{\tau_{1}\times{Abs}_{P_{1,L}}+\tau_{2}\times{Abs}_{P_{2,L}}+\tau_{3}\times{Abs}_{P_{3,L}}}{\tau_{0}+\tau_{1}+\tau_{2}+\tau_{3}+\tau_{4}}=\frac{\tau_{1}\times{Abs}_{P_{1,L}}+\tau_{2}\times{Abs}_{P_{2,L}}+\tau_{3}\times{Abs}_{P_{3,L}}}{\frac{1}{117.5\times F_{c}}+\tau_{1}+\tau_{2}+\tau_{3}+\tau_{4}}$$

(Equation 1)

**Flash photolysis measurement to demonstrate interactions between the *Gt*ACR1 rhodopsin domain and the CPD.**

Flash photolysis measurements were performed to demonstrate interactions between the *Gt*ACR1 rhodopsin domain and the CPD using the same experimental methods described above. We measured the transient absorption of the *Gt*ACR1_ΔCPD with a N-terminal His_6_-tag resuspended in 10 mM HEPES buffer (pH 7.5) containing 1 M NaCl and 0.05% DDM. To demonstrate potential interactions, the CPD resuspended in the same buffer solution was added to the *Gt*ACR1_ΔCPD with the N-terminal His_6_-tag sample at a molar ratio of 1:10.

1. **Supporting Figures**


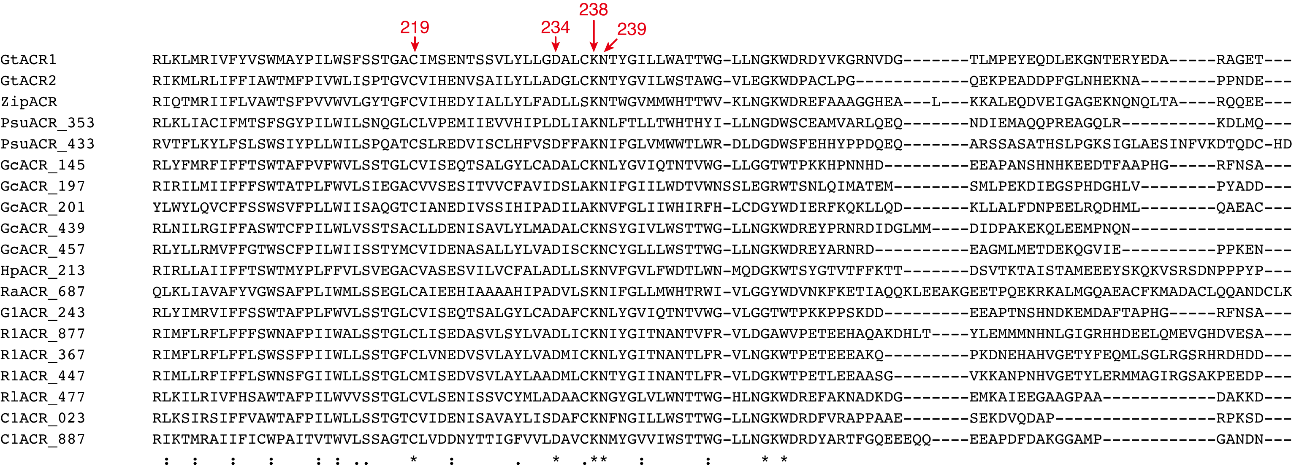

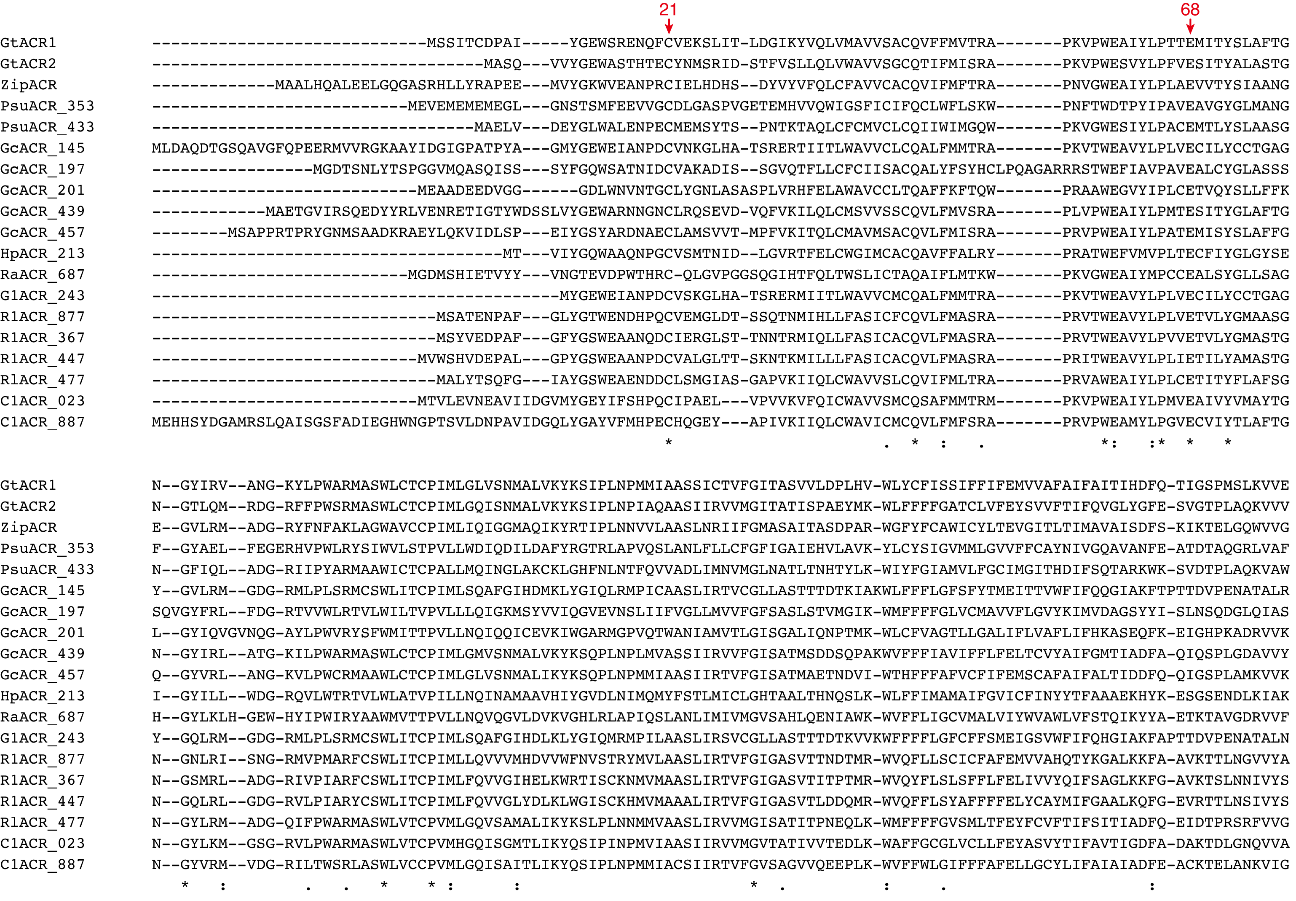
**Supporting Figure S1.**

**Supporting Figure S1. Amino acid sequence alignment of the rhodopsin domain of various ACRs.** The sequences were obtained from several databases reported previously (1, 12, 13). The alignment was analyzed by the MUSCLE program (14, 15). Asterisks (*), colons (:) and dots (.) indicate identical, highly similar and similar, respectively.

**Supporting Figure S2.**


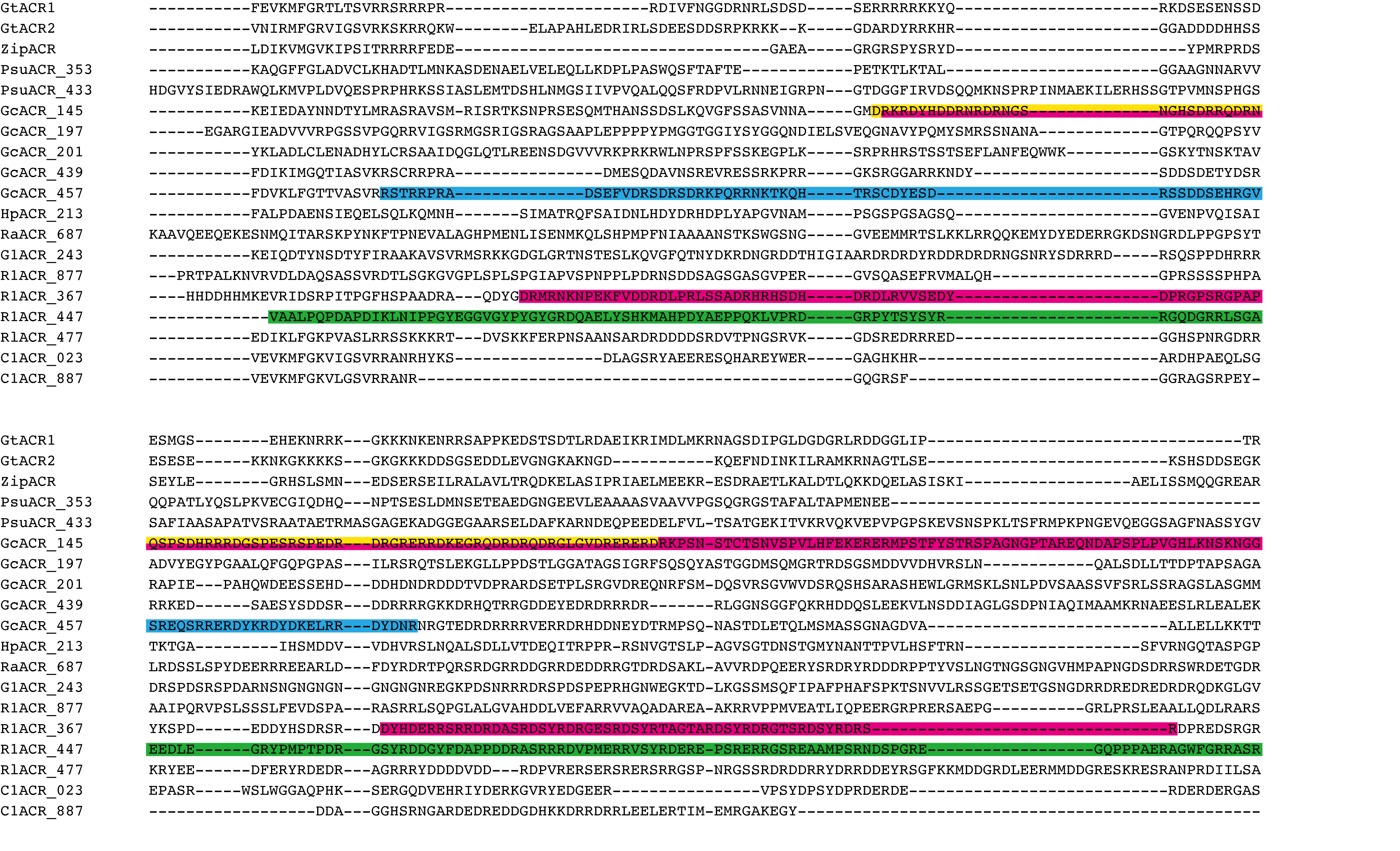


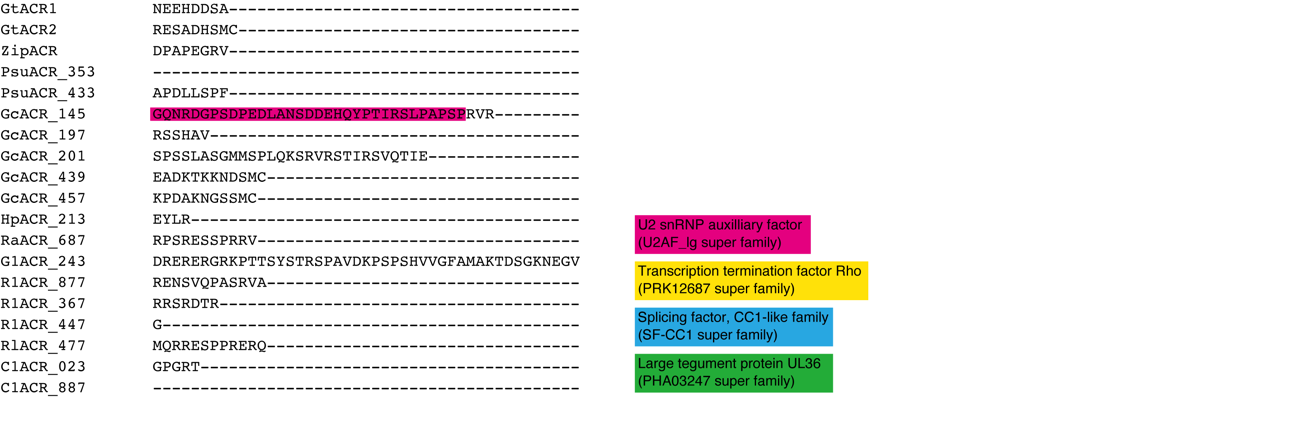


**Supporting Figure S2. Amino acid sequence alignment of the cytoplasmic domain (CPD) of various ACRs.** The sequences were obtained from several databases reported previously (1, 12, 13). The alignment was analyzed by the MUSCLE program (14, 15). Specific domains were analyzed using CD-Search provided by NCBI (16) and highlighted by color.

**Supporting Figure S3.**


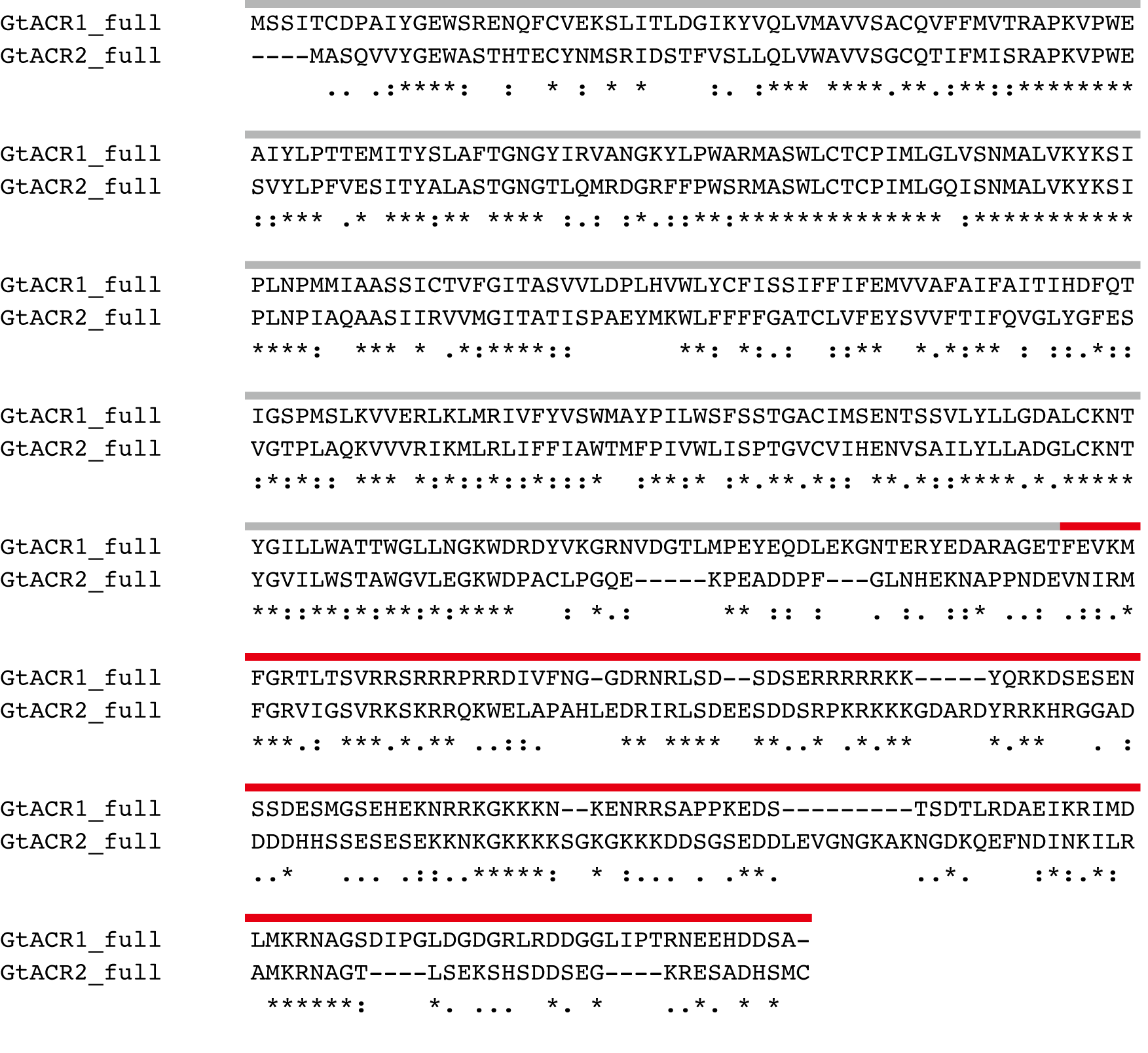


**Supporting Figure S3. Amino acid sequence alignment of the full-length sequences of *Gt*ACR1 and *Gt*ACR2.** The alignment was analyzed by the MUSCLE program (14, 15). Asterisks (*), colons (:) and dots (.) indicate identical, highly similar and similar, respectively. Gray and red bars above the sequences indicate the rhodopsin domain and the CPD, respectively.

**Supporting Figure S4.**


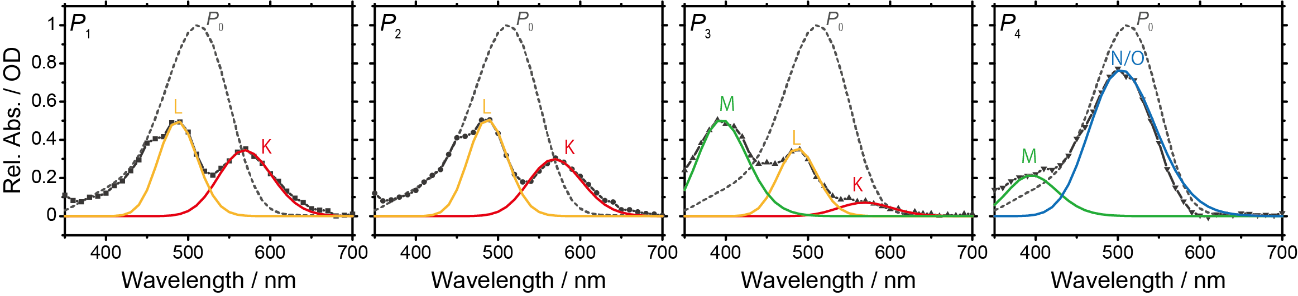


**Supporting Figure S4. Spectral separation of the *P*_i_ states (*i* = 0 – 4) of *Gt*ACR1_full in the presence of Cl^-^.** The separation was performed by Skewed Gaussian fitting as reported previously (8, 9). The names of photo-intermediates are described in each spectrum. *P*_0_ is the pure spectrum in the initial state.

**Supporting Figure S5.**


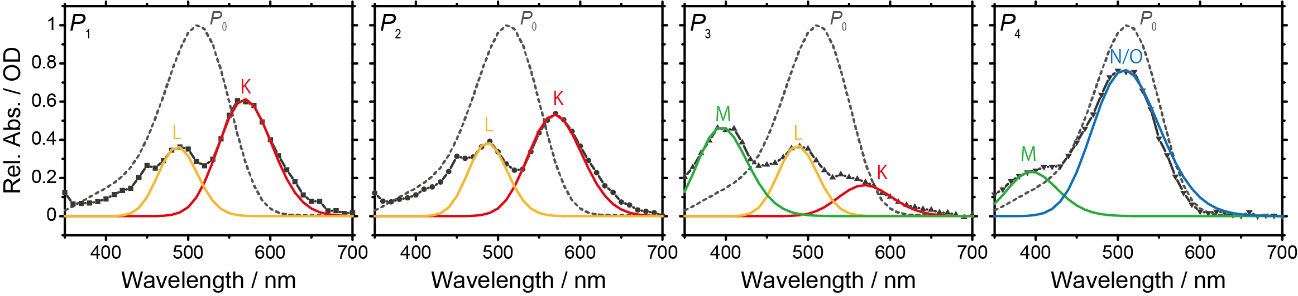


**Supporting Figure S5. Spectral separation of the *P*_i_ states (*i* = 0 – 4) of *Gt*ACR1_ΔCPD in the presence of Cl^-^.** The separation was performed by Skewed Gaussian fitting as reported previously (8, 9). The names of photo-intermediates are described in each spectrum. *P*_0_ is the pure spectrum in the initial state.

**Supporting Figure S6.**


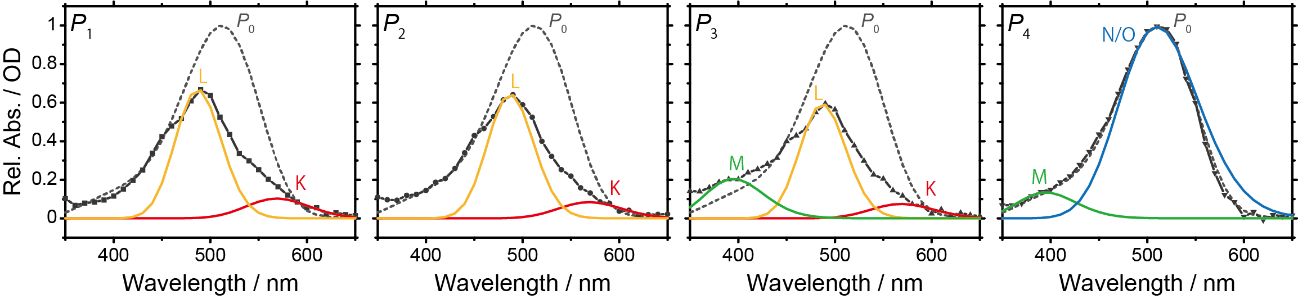


**Supporting Figure S6. Spectral separation of the *P*_i_ states (*i* = 0 – 4) of *Gt*ACR1_full in the presence of NO_3_^-^.** The separation was performed by Skewed Gaussian fitting as reported previously (8, 9). The names of photo-intermediates are described in each spectrum. *P*_0_ is the pure spectrum in the initial state.

**Supporting Figure S7.**


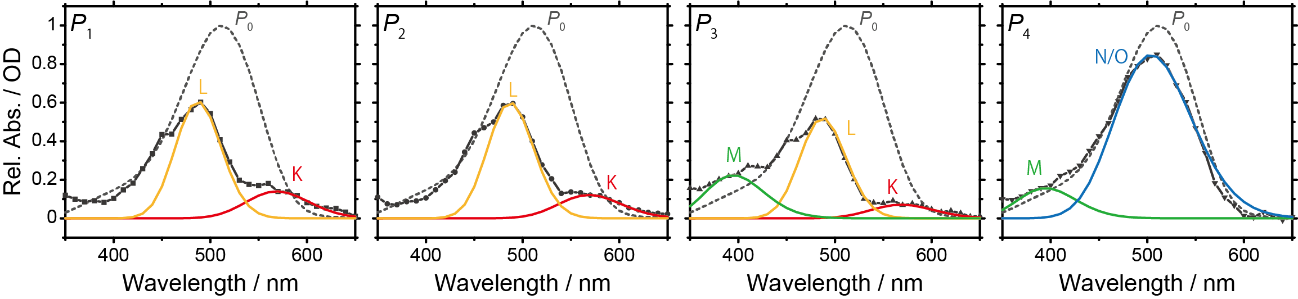


**Supporting Figure S7. Spectral separation of the *P*_i_ states (*i* = 0 – 4) of *Gt*ACR1_ΔCPD in the presence of NO_3_^-^.** The separation was performed by Skewed Gaussian fitting as reported previously (8, 9). The names of photo-intermediates are described in each spectrum. *P*_0_ is the pure spectrum in the initial state.

**Supporting Figure S8.**

**
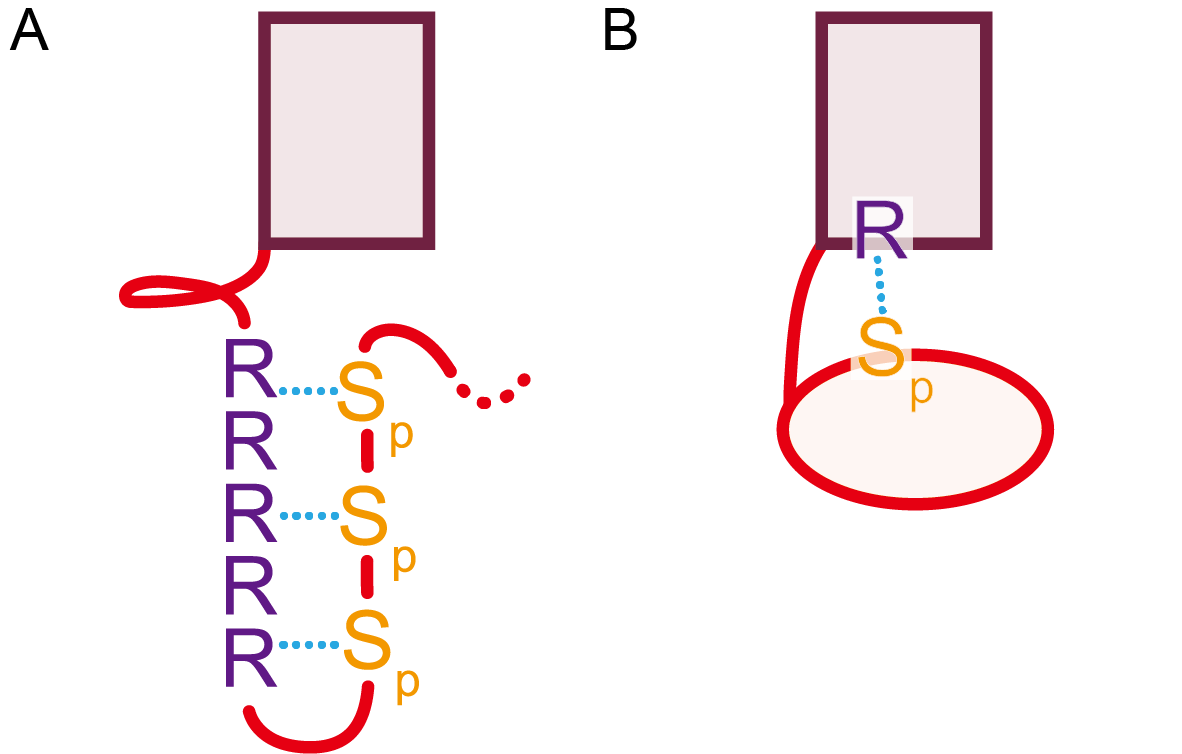
**

**Supporting Figure S8. Schematics of covalent-like electrostatic interactions between Arg and phosphorylated Ser/Thr residues.** This interaction would stabilize the folding structure of the CPD (A) and the interaction between the CPD and the cytoplasmic surface of the rhodopsin domain (B). The interaction between Arg and phosphorylated Ser/Thr is according to (17).

**Supporting Figure S9.**


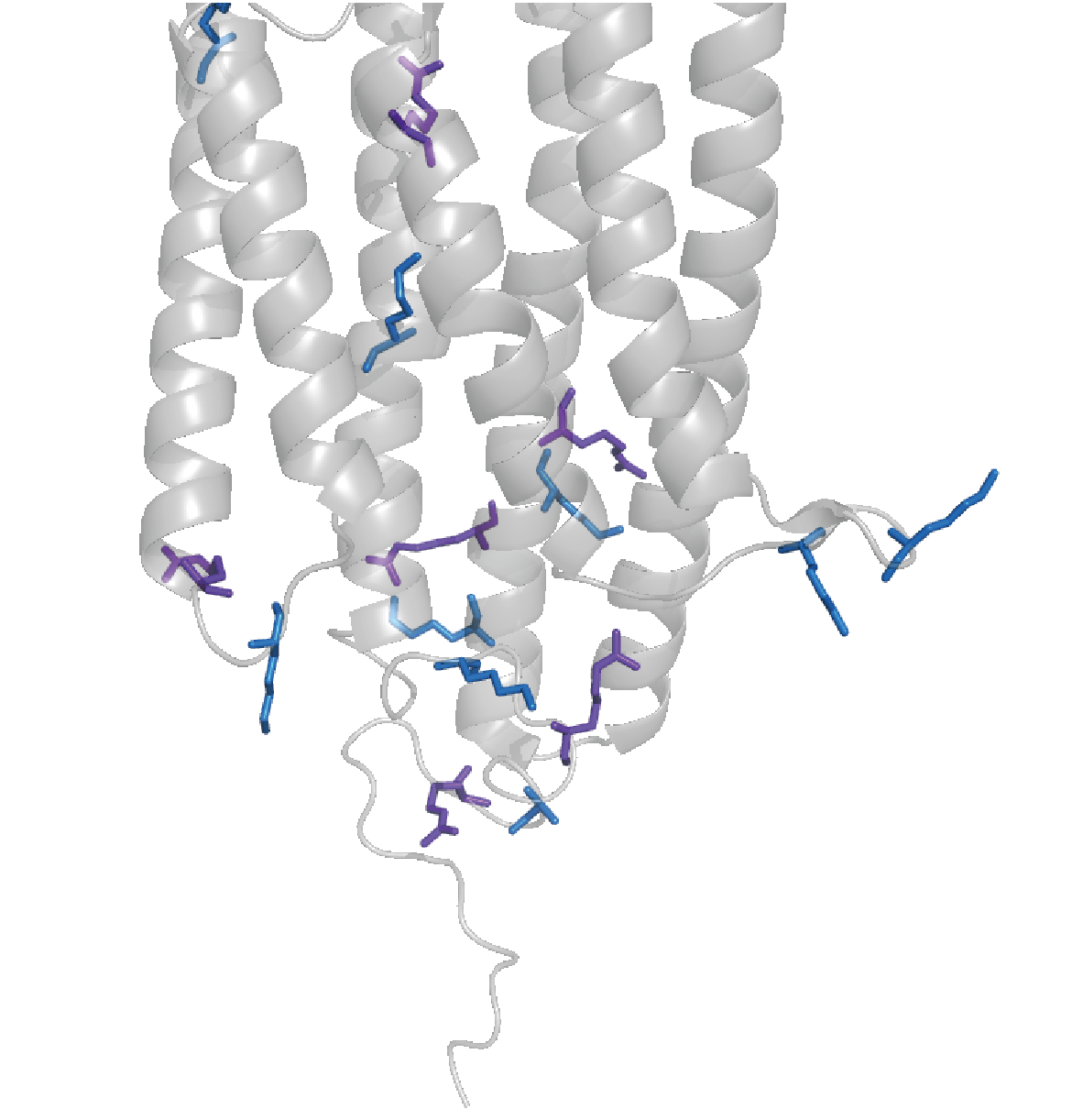


**Supporting Figure S9. The structure of *Gt*ACR1 (PDB ID: 6CSM) (18) pictured by PyMOL.** The cytoplasmic side of the structure is enlarged. Basic amino acids (Lys shown in blue, Arg shown in purple) are localized at the cytoplasmic side.

**Supporting Figure S10.**

**
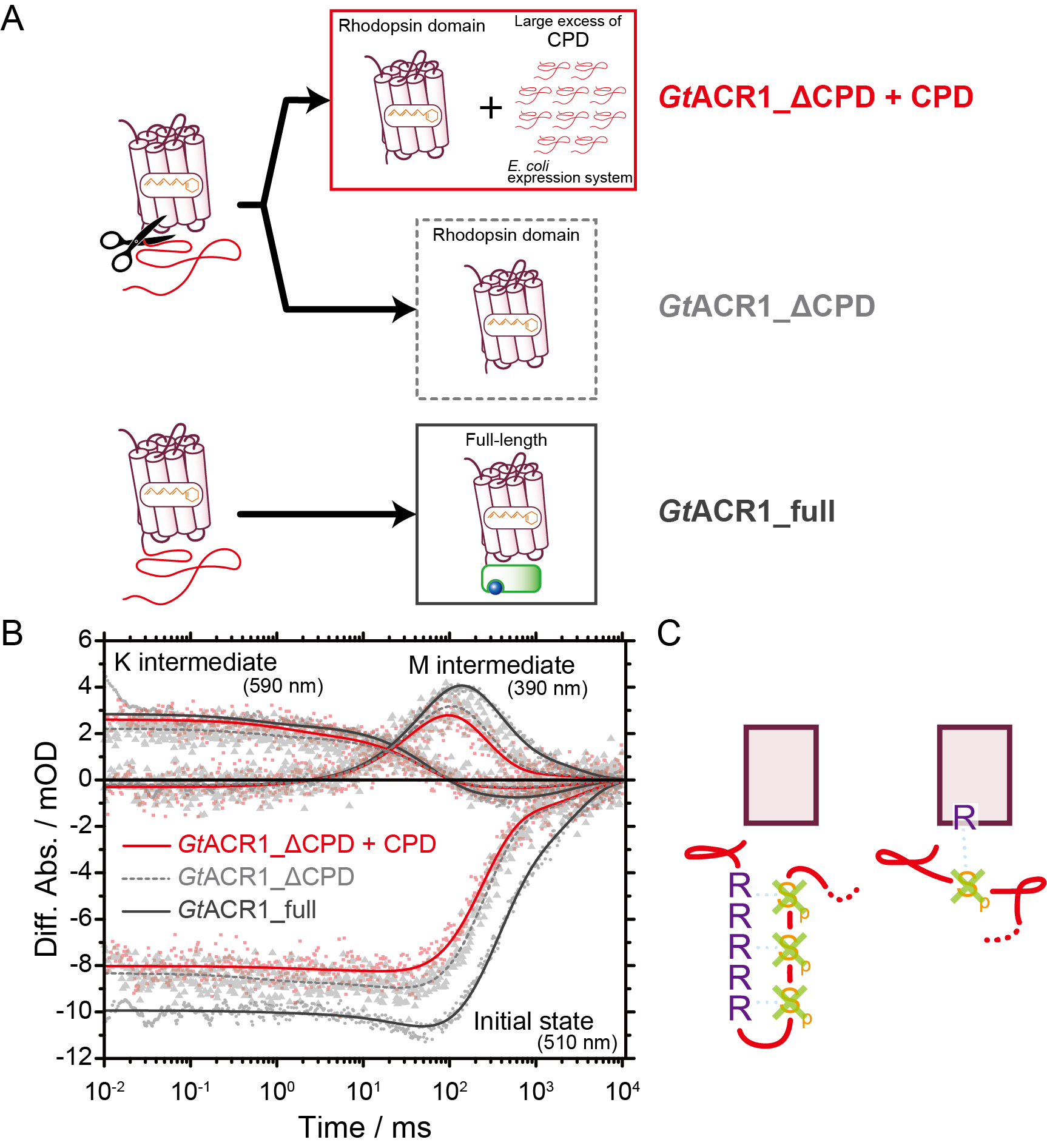
**

**Supporting Figure S10. Demonstration of the interaction between the CPD and the rhodopsin domain of *Gt*ACR1_ΔCPD using flash photolysis.** (A) Illustrations of the experimental procedures used for flash photolysis. (B) Transient absorption changes at 510 nm (initial state), 590 nm (K-intermediate) and 390 nm (M-intermediate) for *Gt*ACR1_ΔCPD and the CPD at a molar ratio of 1:10 (shown in red), *Gt*ACR1_ΔCPD alone (shown in grey) and *Gt*ACR1_full (shown in black). (C) Illustrations showing possible causes for the failure of the experiment.

**Supporting Figure S11.**

**
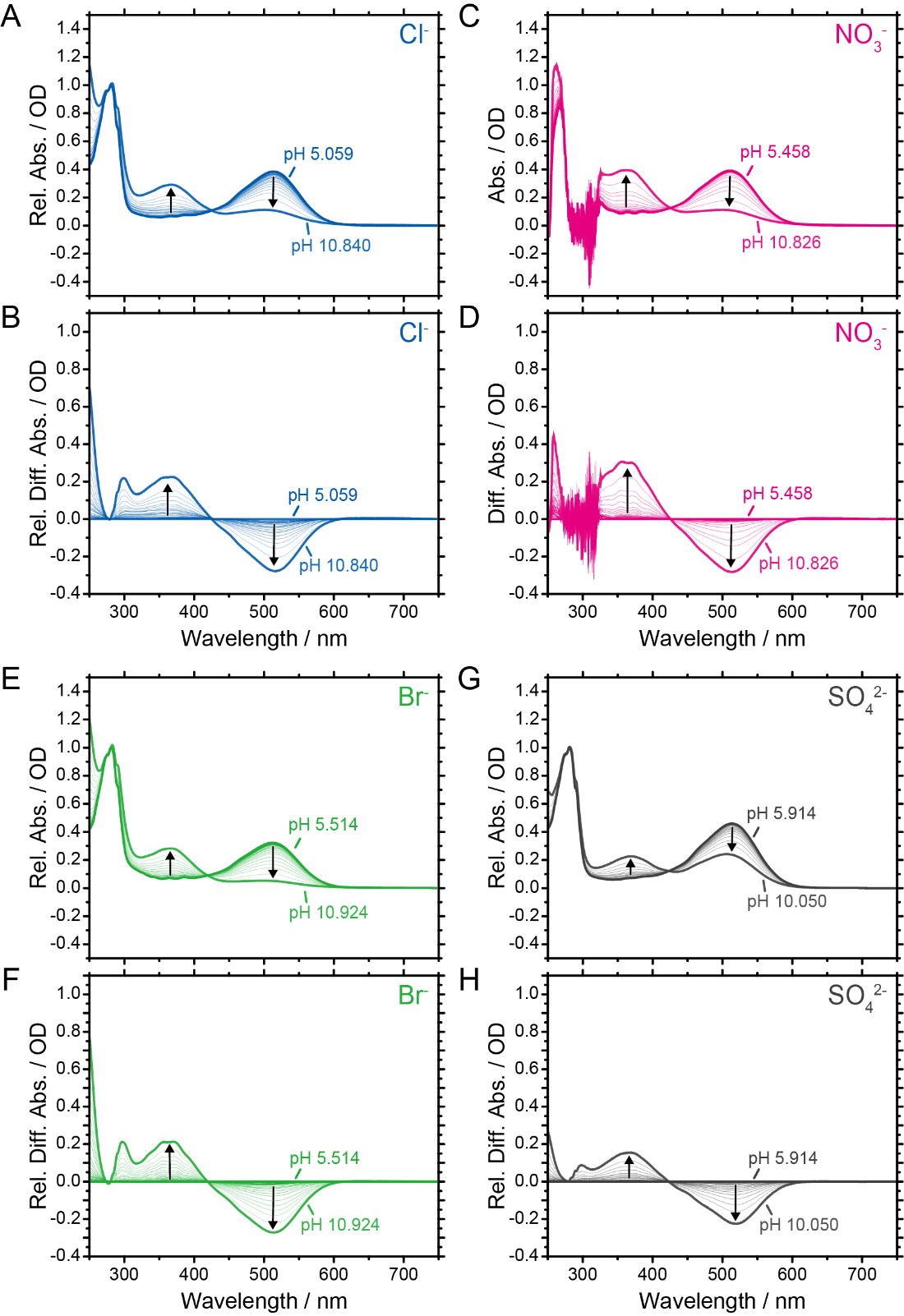
**

**Supporting Figure S11. pH titration of the UV-visible absorption spectra of *Gt*ACR1_ΔCPD.** The UV-visible absolute spectra and difference spectra in the presence of (A, B) 1 M NaCl, (C, D) 1 M NaNO_3_, (E, F) 1 M NaBr and (G, H) 333.3 mM Na_2_SO_4_, respectively. Due to near-UV absorption by NO_3_^-^, the spectra are significantly noisy.

1. **References**

1. Govorunova, E. G., Sineshchekov, O. A., Janz, R., Liu, X., and Spudich, J. L. (2015) NEUROSCIENCE. Natural light-gated anion channels: A family of microbial rhodopsins for advanced optogenetics. *Science*. **349**, 647–50

2. Tsukamoto, T., Kikuchi, C., Suzuki, H., Aizawa, T., Kikukawa, T., and Demura, M. (2018) Implications for the impairment of the rapid channel closing of Proteomonas sulcata anion channelrhodopsin 1 at high Cl- concentrations. *Sci. Rep.* **8**, 13445

3. Kikuchi, C., Kurane, H., Watanabe, T., Demura, M., Kikukawa, T., and Tsukamoto, T. (2021) Preference of Proteomonas sulcata anion channelrhodopsin for NO3- revealed using a pH electrode method. *Sci. Rep.* **11**, 7908

4. Doi, Y., Watanabe, J., Nii, R., Tsukamoto, T., Demura, M., Sudo, Y., and Kikukawa, T. (2022) Mutations conferring SO42- pumping ability on the cyanobacterial anion pump rhodopsin and the resultant unique features of the mutant. *Sci. Rep.* **12**, 16422

5. Kushnirov, V. V. (2000) Rapid and reliable protein extraction from yeast. *Yeast*. **16**, 857–60

6. Rasband, W. S. ImageJ. *U.S. Natl. Institutes Heal. Bethesda, Maryland, U.S.A.*

7. Kikukawa, T., Kusakabe, C., Kokubo, A., Tsukamoto, T., Kamiya, M., Aizawa, T., Ihara, K., Kamo, N., and Demura, M. (2015) Probing the Cl--pumping photocycle of pharaonis halorhodopsin: Examinations with bacterioruberin, an intrinsic dye, and membrane potential-induced modulation of the photocycle. *Biochim. Biophys. Acta*. **1847**, 748–58

8. Hasegawa, C., Kikukawa, T., Miyauchi, S., Seki, A., Sudo, Y., Kubo, M., Demura, M., and Kamo, N. (2007) Interaction of the halobacterial transducer to a halorhodopsin mutant engineered so as to bind the transducer: Cl- circulation within the extracellular channel. *Photochem. Photobiol.* **83**, 293–302

9. Sato, M., Kubo, M., Aizawa, T., Kamo, N., Kikukawa, T., Nitta, K., and Demura, M. (2005) Role of putative anion-binding sites in cytoplasmic and extracellular channels of Natronomonas pharaonis halorhodopsin. *Biochemistry*. **44**, 4775–84

10. Sineshchekov, O. A., Li, H., Govorunova, E. G., and Spudich, J. L. (2016) Photochemical reaction cycle transitions during anion channelrhodopsin gating. *Proc. Natl. Acad. Sci. U. S. A.* **113**, E1993-2000

11. Dreier, M.-A., Althoff, P., Norahan, M. J., Tennigkeit, S. A., El-Mashtoly, S. F., Lübben, M., Kötting, C., Rudack, T., and Gerwert, K. (2021) Time-resolved spectroscopic and electrophysiological data reveal insights in the gating mechanism of anion channelrhodopsin. *Commun. Biol.* **4**, 578

12. Govorunova, E. G., Sineshchekov, O. A., Rodarte, E. M., Janz, R., Morelle, O., Melkonian, M., Wong, G. K. S., and Spudich, J. L. (2017) The Expanding Family of Natural Anion Channelrhodopsins Reveals Large Variations in Kinetics, Conductance, and Spectral Sensitivity. *Sci. Rep.* **7**, 43358

13. Govorunova, E. G., Sineshchekov, O. A., Hemmati, R., Janz, R., Morelle, O., Melkonian, M., Wong, G. K. S., and Spudich, J. L. (2018) Extending the Time Domain of Neuronal Silencing with Cryptophyte Anion Channelrhodopsins. *eNeuro*. **5**, 1–16

14. Edgar, R. C. (2004) MUSCLE: Multiple sequence alignment with high accuracy and high throughput. *Nucleic Acids Res.* **32**, 1792–1797

15. Edgar, R. C. (2004) MUSCLE: a multiple sequence alignment method with reduced time and space complexity. *BMC Bioinformatics*. **5**, 113

16. Marchler-Bauer, A., and Bryant, S. H. (2004) CD-Search: protein domain annotations on the fly. *Nucleic Acids Res.* **32**, W327-31

17. Woods, A. S., and Ferré, S. (2005) Amazing stability of the arginine-phosphate electrostatic interaction. *J. Proteome Res.* **4**, 1397–402

18. Kim, Y. S., Kato, H. E., Yamashita, K., Ito, S., Inoue, K., Ramakrishnan, C., Fenno, L. E., Evans, K. E., Paggi, J. M., Dror, R. O., Kandori, H., Kobilka, B. K., and Deisseroth, K. (2018) Crystal structure of the natural anion-conducting channelrhodopsin GtACR1. *Nature*. **561**, 343–348
